# CD47 interactions with exportin-1 limit the targeting of m^7^G-modified RNAs to extracellular vesicles

**DOI:** 10.1007/s12079-021-00646-y

**Published:** 2021-11-29

**Authors:** Sukhbir Kaur, Alejandra Cavazos Saldana, Abdel G. Elkahloun, Jennifer D. Petersen, Anush Arakelyan, Satya P. Singh, Lisa M. Jenkins, Bethany Kuo, Bianca Reginauld, David G. Jordan, Andy D. Tran, Weiwei Wu, Joshua Zimmerberg, Leonid Margolis, David D. Roberts

**Affiliations:** 1 Laboratory of Pathology, Center for Cancer Research, National Cancer Institute, National Institutes of Health, Building 10 Room 2S235, 10 Center Dr, Bethesda, MD 20892-1500 USA; 2 Cancer Genetics Branch, National Human Genome Research Institute, National Institutes of Health, Bethesda, USA; 3 Section On Integrative Biophysics, Division of Basic and Translational Biophysics, Eunice Kennedy-Shriver National Institute of Child Health and Human Development, National Institutes of Health, Bethesda, USA; 4 Section On Intercellular Interactions, Division of Basic and Translational Biophysics, Eunice Kennedy-Shriver National Institute of Child Health and Human Development, National Institutes of Health, Bethesda, USA; 5 Inflammation Biology Section, Laboratory of Molecular Immunology, National Institute of Allergy and Infectious Diseases, National Institutes of Health, Bethesda, USA; 6 Laboratory of Cell Biology, Center for Cancer Research, National Cancer Institute, National Institutes of Health, Bethesda, USA; 7 Confocal Microscopy Core Facility, Center for Cancer Research, National Cancer Institute, National Institutes of Health, Bethesda, USA

**Keywords:** Extracellular vesicles, Exportin-1, RNA trafficking, CD47, Micro-RNA, Ubiquilin-1

## Abstract

CD47 is a marker of self and a signaling receptor for thrombospondin-1 that is also a component of extracellular vesicles (EVs) released by various cell types. Previous studies identified CD47-dependent functional effects of T cell EVs on target cells, mediated by delivery of their RNA contents, and enrichment of specific subsets of coding and noncoding RNAs in CD47^+^ EVs. Mass spectrometry was employed here to identify potential mechanisms by which CD47 regulates the trafficking of specific RNAs to EVs. Specific interactions of CD47 and its cytoplasmic adapter ubiquilin-1 with components of the exportin-1/Ran nuclear export complex were identified and confirmed by coimmunoprecipitation. Exportin-1 is known to regulate nuclear to cytoplasmic trafficking of 5’-7-methylguanosine (m^7^G)-modified microRNAs and mRNAs that interact with its cargo protein EIF4E. Interaction with CD47 was inhibited following alkylation of exportin-1 at Cys^528^ by its covalent inhibitor leptomycin B. Leptomycin B increased levels of m^7^G-modified RNAs, and their association with exportin-1 in EVs released from wild type but not CD47-deficient cells. In addition to perturbing nuclear to cytoplasmic transport, transcriptomic analyses of EVs released by wild type and CD47-deficient Jurkat T cells revealed a global CD47-dependent enrichment of m^7^G-modified microRNAs and mRNAs in EVs released by CD47-deficient cells. Correspondingly, decreasing CD47 expression in wild type cells or treatment with thrombospondin-1 enhanced levels of specific m^7^G-modified RNAs released in EVs, and re-expressing CD47 in CD47-deficient T cells decreased their levels. Therefore, CD47 signaling limits the trafficking of m^7^G-modified RNAs to EVs through physical interactions with the exportin-1/Ran transport complex.

**Supplementary Information:**

The online version contains supplementary material available at 10.1007/s12079-021-00646-y.

## Introduction

Extracellular vesicles (EVs) ranging in size from 30 to 500 nm are formed either by budding from the plasma membrane or via multivesicular bodies (MVBs) that release EVs upon fusion with the plasma membrane (Thery et al. 2009). EVs can modulate responses in recipient cells by transferring their cargo, including mRNAs, miRNAs and other non-coding RNAs (Hergenreider et al. 2012; Mittelbrunn and Sanchez-Madrid 2012; Kaur et al. 2018b). This intercellular communication mediated by EVs has physiological functions and is involved in the pathogenesis of diseases including cancer, Alzheimer’s and autoimmune diseases (Anderson et al. 2016; Gillet et al. 2016; Kumar and Reddy 2016; Tan et al. 2016).

CD47 is a signaling receptor for the secreted extracellular matrix protein thrombospondin-1 (TSP1) and the counter-receptor for signal regulatory protein-α (Barclay and Van den Berg 2014, Soto-Pantoja et al. 2015). CD47 signaling induced by TSP1 regulates a variety of cellular responses including nitric oxide/cGMP signaling, autophagy, stem cell self-renewal, and metabolic responses of cells to stress (Soto-Pantoja et al. 2015). In endothelial cells CD47 signaling inhibits angiogenesis (Kaur et al. 2010; Soto-Pantoja et al. 2015). TSP1 signaling mediated by CD47 in T cells globally inhibits TCR-mediated activation (Li et al. 2001, 2002; Kaur et al. 2011; Miller et al. 2013), regulates differentiation of Th17 and regulatory T cells (Van et al. 2008; Rodriguez-Jimenez et al. 2019), and limits T cell-dependent inflammatory responses in vivo (Lamy et al. 2007). CD47 signaling in human and murine cytotoxic T cells inhibits their antigen-dependent killing of target tumor cells in vitro (Soto-Pantoja et al. 2014; Schwartz et al. 2019). Conversely, eliminating CD47 or TSP1 in the tumor microenvironment enhances the control of tumor growth by local tumor irradiation in immune competent but not in T cell-deficient mice or following depletion of CD8 T cells (Isenberg et al. 2008; Soto-Pantoja et al. 2014). Systemic suppression of CD47 expression enhances tumor ablation when combined with ionizing radiation, adoptive T cell transfer, chemotherapy, or a CTLA4 checkpoint inhibitor (Maxhimer et al. 2009; Soto-Pantoja et al. 2014; Feliz-Mosquea et al. 2018; Schwartz et al. 2019).

CD47 is present on EVs isolated from various cell types and biological fluids (Holovati et al. 2008; Sadallah et al. 2011; Rho et al. 2013; Kaur et al. 2014b), but its role in EV function remains unclear. CD47 is one of 22 proteins recently identified to be consistently enriched in exosomes across a variety of cell types (Kugeratski et al. 2021). Our recent studies indicate that CD47 regulates both the RNA composition of EVs released by cells and their effects on the target cells that take up these EVs. Treatment of CD47^−^ Jurkat T cells with EVs released by wild type (WT) cells restored regulation of their activation by TSP1, whereas treatment of WT cells with EVs from CD47^−^ cells conferred resistance to regulation of their activation by TSP1 (Kaur et al. 2014b). Differences in the RNA contents of EVs released from WT and CD47^−^ cells were consistent with their delivery of coding and noncoding RNAs and the CD47-dependent ability of these EVs to modulate of gene expression and signaling in recipient T cells and vascular endothelial cells, but the relevant CD47-dependent regulatory RNAs remained to be defined. Treatment with EVs from WT and CD47^−^ T cells differentially regulated expression of multiple mRNAs in recipient endothelial cells including CD69 and TNFα, and siRNA-mediated silencing of CD47 in the donor WT T cells ablated the CD47-dependent activity of EVs released from these cells to regulate CD69 and TNFα mRNA expression in recipient endothelial cells. Treatment with a function-blocking CD47 antibody did not inhibit the uptake by endothelial cells of EVs derived from triple negative breast cancer stem cells, but the antibody altered the resulting changes in endothelial cell gene expression, VEGFR2 signaling, and endothelial-mesenchymal transition induced by these EVs (Kaur et al. 2014b, 2018b). We further found that CD47^+^ EVs released by Jurkat T cells contain non-coding RNAs, including miRNAs, that are distinct from those in CD63^+^ and MHCI^+^ EVs released by the same cells (Kaur et al. 2018a), suggesting that the packaging of RNAs into specific subpopulations of EVs is regulated by CD47.

Here we examined the mechanisms by which CD47 directly or indirectly regulates which RNAs are packaged into T cell EVs. CD47 contains 5 membrane spanning segments followed by a short cytoplasmic tail that lacks interactions with known signaling molecules except for binding to ubiquilin-1, which mediates CD47 interactions with heterotrimeric G proteins and regulates their signaling in some cells (Wu et al. 1999; N'Diaye and Brown 2003). We show that CD47 expression and signaling induced by its ligand TSP1 control the exportin-1/CRM1-dependent microRNA biogenesis pathway. Mediated by physical interactions that control the subcellular localization of exportin-1 (encoded by *XPO1*), the CD47/ubiquilin-1 complex regulates intracellular trafficking of capped miRNAs and mRNAs and their trafficking into EVs. These results establish TSP1/CD47 signaling as a regulator of nuclear/cytoplasmic RNA trafficking and the subsequent packaging and release of a subset of 5’-7-methylguanosine-modified (m^7^G) RNAs in EVs.

## Materials and methods

### Cell culture and reagents

WT Jurkat T cells and the JinB8 (CD47^−^) somatic mutant (Reinhold et al. 1999) were cultured in a 5% CO_2_ atmosphere in RPMI 1640 medium with 10% FBS, penicillin, and streptomycin at 37 °C. The cells were used for study within 4–8-weeks of continuous culture. Murine CD3^+^ T cells and plasma were isolated from WT and *Cd47*^*−/−*^ C57Bl/6 J mice (The Jackson Laboratory) (Lindberg et al. 1996). Mice were maintained in strict accordance with the recommendations in the Guide for the Care and Use of Laboratory Animals of the National Institutes of Health under protocol LP-012 approved by the Animal Care and Use Committee of the National Cancer Institute.

WT Jurkat T cells were transfected with CRISPR/Cas9 using a CD47 gRNA PC200.Hcd47.g3a (CTACTGAAGTATACGTAAG ngg), as targeting sequence (Washington University Genome Engineering Center). CD47-CRISPR (1 µg) was transiently transfected using an AMAXA kit from Lonza. After 24 h, the transfected cells were transferred to HITES medium, and after incubation at 37°, EVs were harvested using the Exo-quick kit (SBI system Biosciences). The downregulation of CD47 expression was confirmed using flow cytometry analysis (data not shown). Total RNA from cells and EVs were prepared using the miRNA easy kit from QIAGEN (Hilden, Germany), and real time PCR was performed using pri-miRNA primers (Qiagen).

The CD47 (Isoform 2)-mClover-DHFR fusion was cloned as follows: CD47-EYFP fragment was cloned using Xho1 and BamH1 sites in the mClover2-N1 plasmid. The dihydrofolate reductase-derived destabilizing domain carrying the mutations (R12H), (N18T), (V19A) and (G67S) was amplified from pBMN YFP-DHFR(DD) (Iwamoto et al. 2010) and cloned using BsrG1 and Xba1 sites of mClover2-N1 plasmid. To re-express CD47, JinB8 T cells were transfected with 1 µg of control vector and CD47 plasmids using an Amaxa Kit from Lonza. The transfected cells and their EVs were harvested from transiently transfected or stable cell lines that were generated using G418 selection. Total RNA was harvested using the miRNA Easy kit from Qiagen.

### EV characterization and validation

Minimal Information for Studies of Extracellular Vesicles criteria (Thery et al. 2018) were used to compare EVs isolated as reported previously using Exo-quick (SBI) to identify CD47-dependent mRNAs (Kaur et al. 2014b) with EVs isolated for some of the present studies using size exclusion and ultracentrifugation. Isolation protocols and analytical methods are detailed in the Extended Methods. Negative stain electron microscopy demonstrated similar morphologies (Figure S1A-D), and NanoSight analysis demonstrated similar size distributions (Figure S1E-F). Similar expression of the EV protein markers CD63, CD81, CD9, and CD41 on EVs from WT and CD47^−^ cells purified by size exclusion chromatography were determined using NanoView (Figure S1G-L). Lack of surface and intracellular CD47 expression in the CD47^−^ mutant was verified using flow cytometry analysis (Figure S1M). Although several EV purification methods were used, these data confirm consistent properties of EVs isolated from WT and CD47^−^ Jurkat T cells using each method.

### RNA extraction and Real-Time PCR

MicroRNA and total RNA were extracted from WT and CD47^−^ T cell-derived EVs (isolated and purified as indicated in extended methods) using the miRNA easy kit from QIAGEN. The concentration and RNA quality were measured using a RNA-Bio analyzer. 100–200 ng of total RNA was used for first strand miRNA synthesis (Quanta Bioscience) as described (Kaur et al. 2016). First strand cDNA synthesis used a Maxima kit (2-Step RT PCR, Thermo Scientific) according to the manufacturer’s instructions or from Maxima first strand (Thermo scientific Fisher). U6 was used as a reference control gene for real -time PCR analysis as indicated in the legends. Cytoplasmic and nuclear RNA were extracted using the Cytoplasmic and Nuclear RNA Purification Kit from Norgen BioTek Corporation (Thorold ON). RNA was extracted according to manufacturer’s instructions. CD3^+^ cells from WT and *Cd47* null mice (n = 3) were purified using Pan T Cell Isolation Kit II, mouse from Miltenyi Biotec, and mRNA isolation and real-time PCR were performed as described above using primers listed in Figure S7.

### Microarray analysis

Total EVs from WT and CD47^−^ T cells (n = 3) were collected as previously described (Kaur et al. 2014b), and miRNA microarray analysis performed as described previously (Kaur et al. 2018a). Plasma was prepared from blood obtained by cardiac puncture from WT and *Cd47* null mice. Plasma samples were pre-treated with thrombin before using the standard ExoQuick kit from SBI for EV extraction, and total miRNAs were extracted using the QIAGEN kit as described previously (Kaur et al. 2018a). All the data has been submitted at https://www.ncbi.nlm.nih.gov/geo/query/acc.cgi?acc=GSE132646.

### miRNA sequencing analysis

WT and CD47^−^ Jurkat T cells and their EVs were subjected to miRNA sequencing using the Qiagen miRNA kit and sequenced on one NextSeq run with 76 bases single end reads. at the Sequencing Core Facility, Frederick National Laboratory for Cancer Research, National Cancer Institute. All of the samples had 20–31 million reads. About 96% of pass filter reads had a score of Q30 or above. The miRNA sequencing analysis was performed using default settings of the CLC Genomics Workbench with Biomedical Genomics Analysis plugin and the Quantify miRNA expression with the QIAseq miRNA Quantification ready-to-use workflow. The mature and seed/precursors miRNAs were visualized using the Create Combined Report tool and the QIA seq miRNA Differential Expression workflow. All the data are deposited at GEO accession GSE168187: Go to https://www.ncbi.nlm.nih.gov/geo/query/acc.cgi?acc=GSE168187. Venn diagrams depicting CD47-dependent mature and seed/precursor miRNAs that were significant at *p* < 0.05 in cells and EVs were created via ‘FunRich3.1.3 from http://www.funrich.org/.

### Confocal microscopy

WT and CD47^−^ T cells were plated on poly-lysine coated 8-well Lab Tek chamber slides. The cells were fixed and immunostained as previously described (Kaur et al. 2014a) using rabbit anti-human CD47, mouse anti-human CD47, rabbit anti-human exportin-1 (Proteintech), human CD47 antibody B6H12, exportin-1/CRM1 from Santa Cruz Biotechnologies, RANBP1, RANGAP1 from Cell Signaling. Alexa 488 and Alexa 594 conjugated second antibodies were purchased from Thermo Scientific Fisher. The images were captured using a Zeiss LSM 780 microscope with a 63 × objective (Plan-Apochromat, 1.40NA), and image analyses were performed using Zen blue software. The detail of confocal analysis is described in extended methods.

### IP-Western blots

WT and CD47^−^ T cells were lysed using RIPA buffer or NE-PER Nuclear and Cytoplasmic Extraction Kit from Thermo Scientific Fisher according to manufacturer’s instructions as indicated in the figure legends. A BCA protein assay (Pierce) was performed in order to quantify total protein. Prior to IP, the indicated lysates were pre-treated in the presence or absence of 10 µM GTPyS (Sigma-Aldrich) for 15 min on ice. Immunoprecipitation was performed using Super Mag Protein A/G Beads (Ocean NanoTech, San Diego, CA). Beads were incubated with anti-CD47 antibody (5 µg) Proteintech or Biolegend) and rotated for 30 min at room temperature or overnight at 4 °C. Equal volumes of conjugated beads were added to lysed samples (300 µg protein), which where incubated overnight at 4 °C. The next day, the beads were washed two times with PBS and protease inhibitor cocktail, resuspended in mixture of RIPA and LDS buffer, and heated at 95 °C for 5 min. The lysates were then loaded into a Bis–Tris NuPAGE gel (Invitrogen) for Western blotting. Gels were electrophoresed at 200 V and transferred onto polyvinylidene difluoride membranes using Invitrogen iBlot system and transfer stacks. Membranes were blocked with 3–5% BSA for one hour, incubated with primary antibody (1:500) overnight at 4 °C., incubated with secondary antibody (1: 1000) for 1 h at room temperature, and imaged on a Bio-Rad Chemidoc MP system. The detail of western blots analysis is described in extended methods.

### EV immunoprecipitation-western blots

Total EVs were extracted using Buffer A from the Exo-spin Kit and directly subjected to IP precipitation using immunoconjugated super magnetic beads with exportin-1 antibody overnight at 4 °C in PBS Buffer containing Triton X-100. The exportin-1 captured beads were washed using a magnetic rack and were boiled at 95 °C using 1X LDS buffer dye (Lithium dodecyl sulfate, pH 8.4 (Thermo Scientific Fisher). Immunoprecipitation and western blotting were performed using CD47 (Proteintech and Biolegend, San Diego, CA), exportin-1, RANBP1, RANBP3, RANGAP1, ubiquilin-1, CROP, and GAPDH antibodies.

### Mass spectrometry analysis

Immunoprecipitations were separated by gel electrophoresis, and proteins were digested in gel with trypsin as described (Shevchenko et al. 2006). Dried peptides were solubilized in 2% acetonitrile, 0.5% acetic acid, 97.5% water for mass spectrometry analysis. They were trapped on a trapping column and separated on a 75 µm × 15 cm, 2 µm Acclaim PepMap reverse phase column (Thermo Scientific) using an UltiMate 3000 RSLCnano HPLC (Thermo Scientific) coupled online to an Orbitrap Fusion mass spectrometer (Thermo Scientific). Parent full-scan mass spectra were collected in the Orbitrap mass analyzer set to acquire data at 120,000 FWHM resolution; ions were then isolated in the quadrupole mass filter, fragmented within the HCD cell (HCD normalized energy 32%, stepped ± 3%), and the product ions analyzed in the ion trap. Proteome Discoverer 2.1 (Thermo Scientific) was used to search the data against human proteins from the UniProt database using SequestHT. The search was limited to tryptic peptides, with maximally two missed cleavages allowed. Cysteine carbamidomethylation was set as a fixed modification, and methionine oxidation set as a variable modification. The precursor mass tolerance was 10 ppm, and the fragment mass tolerance was 0.6 Da. The Percolator node was used to score and rank peptide matches using a 1% false discovery rate. The data is deposited at ftp://MSV000086969@massive.ucsd.edu.

### RNA immunoprecipitation (RIP)

Anti-exportin-1 (Proteintech) and anti-m^3^G-cap/m^7^G-cap (clone H-20) from Synaptic Systems (Goettingen, Germany) were used for RIP performed using the EZ-Magna RIP™ RNA-Binding Protein Immunoprecipitation Kit (Millipore Sigma, St. Louis, MO) according to the manufacturer’s instructions. Due to negative values for 40–60% fraction of EV, RIP with control antibody using total cellular RNA from WT T cells was used as negative control for normalization.

### Mature and precursor miRNA analysis

Total RNA extracted from WT and CD47^−^ cells and their EVs and used to prepare cDNAs. Real-time PCR was performed according to manufactures instructions using miScript II RT Kit and real-time PCR was performed with miScript SYBR Green PCR Kit (Qiagen). Control primer was used for normalization miScript Precursor Assay and miScript Primer Assay (Qiagen). Mature miRNA and precursor miRNA were analyzed using miScript II RT Kit which contain miScript HiSpec Buffer and miScript HiFlex Buffer for cDNA synthesis along with mature and precursor miScript Primer Assays were purchased from Qiagen. The real-time PCR for miRNA expression of mature (miRNA-320a-3p) and precursor of miRNA-320a were analyzed according to manufactures instructions. Mature 22 nucleotide miRNAs were polyadenylated using poly(A) polymerase and reverse transcribed into cDNA using oligo-dT primers. The oligo-dT primers had a 3' degenerate anchor and a universal tag sequence on the 5' end, allowing amplification of mature miRNA in the real-time PCR step. miScript Primer Assay was used in combination with the miScript SYBR Green PCR Kit, to enable quantification of mature miRNA by real time PCR. The combination of polyadenylation and the universal tag addition ensured that miScript Primer Assays do not detect genomic DNA.

### Precursor miRNA analysis

miScript Precursor Assays consist of a precursor-miRNA–specific forward and reverse primer targeting the stem-loop sequence of the precursor miR-320a. The miScript Universal Primer is not used in this analysis. The stem-loop sequence targeted by the miScript Precursor Assay is present in both primary miRNA (pri-miRNA) and precursor miRNA, therefore miScript Precursor Assays provide quantification of both primary and precursor miRNA simultaneously.

### Electron microscopy with immunogold labeling for CD47

Pre-embedding immunogold labeling of Jurkat T cells and negative stain electron microscopy for EVs was performed as reported previously (Chen et al. 2020)(Arai et al. 1992)(Nair et al. 2013). A detailed procedure is in the extended methods.

### Statistical analysis

Western blot density data was subjected to two-way ANOVA (mixed model) multiple comparison mean regardless of rows and column using default settings. For the western blots, *P* values were reported in GP style; 0.1234(ns) 0332 (*), 0.0021(**),0.0002 (***), < 0.0001 (****) were used**.** Raw Ct value of reference control was subtracted from mRNAs Ct, and Two-Way ANOVA (or Mixed Model) analysis was performed in GraphPad Prism. The parameters were set to make multiple comparisons that compared cell means regardless of rows and columns. We corrected for multiple comparisons using the recommended post-hoc Tukey statistical hypothesis test. The *p*-values are used 0.12 (ns),0.033 (*),0.002 (**) < 0.001 (***) for (Fig. 3c–j). For other, Real-time PCR data were analyzed using ANOVA: Two Factor with replication. Image analysis was performed using t-Test: Two samples were compared assuming equal variance using Excel data analysis. Differential expression of miRNA and Anti-m3G-cap/m^7^G-CAP were analyzed using Microsoft excel default setting of ANOVA: Two-Factor with replication for Real-time PCR. The *P*-value (≤ 0.05) is selected as significant with two Sample and Columns (*) Sample, Columns and Interaction (**) are shown in the graphs. For Supplementary Figure S4, statistically significant value was calculated using ANOVA: Two-Factor with replication by comparing WT resting cells versus activated cells and their EVs, Resting cells versus EVs and activated cells versus activated EVs. Similarly, CD47^−^ T resting cells versus activated cells and their EVs, resting cells versus EVs and activated cells versus activated EVs were compared. Two-Factor with replication for Real-time PCR. The *P*-value (≤ 0.05) is selected as significant with two Sample and Columns (*) Sample, Columns and Interaction (**) are shown in the graphs.

## Results

### CD47 regulates the miRNA content of T cell derived EVs

We previously reported that the mRNA composition of EVs derived from WT Jurkat T cells differs from that of EVs from the CD47-deficient (CD47^−^) Jurkat mutant JinB8 (Kaur et al. 2014b). Further analysis identified many differentially expressed noncoding RNAs (Figure S2A), and most were elevated in EVs from the CD47^−^ cells (Figure S2B). Although treatment of human umbilical vein endothelial cells with EVs derived from WT versus CD47^−^ Jurkat cells differentially regulated the expression of 65 mRNAs in the treated cells (Kaur et al. 2014b), only one of these (ZBTB40) overlapped with the mRNAs that were differentially expressed in the EVs (Figure S2C), This suggested that the observed CD47-dependent regulation of mRNA levels in the endothelial cells probably results from activities of noncoding regulatory RNAs in the EVs rather than direct transfer of mRNAs.

**Fig. 1:**
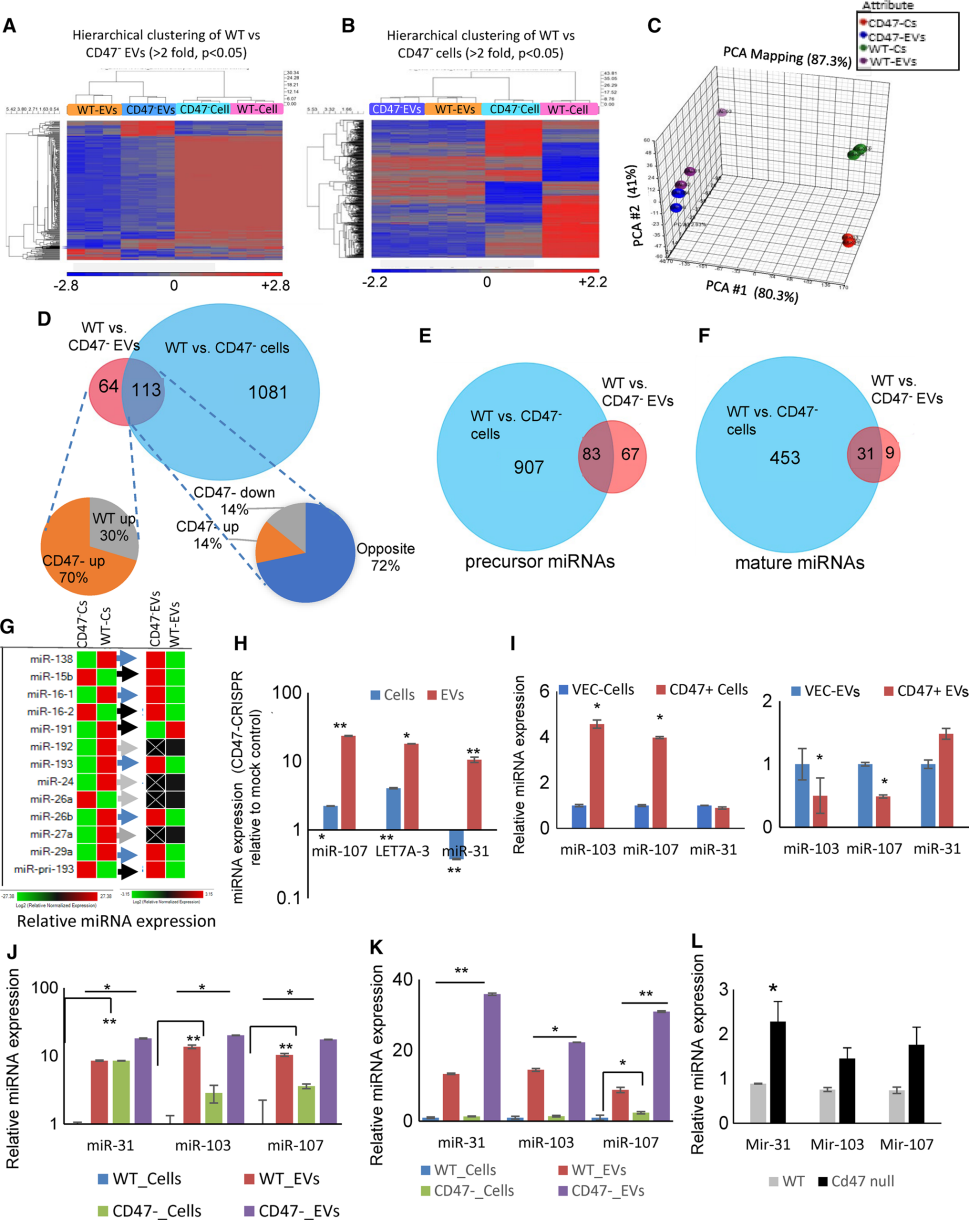
CD47 differentially regulates the abundance of specific miRNAs in cells and EVs. **a** Hierarchical clustering of differentially expressed miRNAs in EVs (> twofold, *p* val < 0.05) with unsupervised clustering of their parental cell data based on microarray analyses of WT and CD47 deficient Jurkat T cells and their released EVs (N = 3) **b** Hierarchical clustering of differentially expressed cellular miRNAs with unsupervised EV data**.** **c** Principal component analysis of miRNA data for WT and CD47^−^ Jurkat T cells and EVs released by these cells. **d** Venn diagram summarizing RNAseq analysis of differentially expressed miRNAs (*p* < 0.05) comparing WT and CD47^−^ cells (Blue) and EVs (Red) (Data S2A, *p* < 0.05). The left pie chart depicts the percentages of miRNAs up- or down-regulated only in CD47^−^ versus WT EVs. The right pie chart presents the percentages of miRNAs up (orange) or down regulated (gray) in CD47^−^ EVs and in cells and miRNAs showing opposite CD47-dependent regulation in cells versus EVs (dark blue). **e, f** Venn diagram presenting numbers of precursor **e** and mature miRNAs **f** differentially expressed in WT vs. CD47^−^ cells (blue) and WT vs CD47^−^ EVs (red) (Data S2A, *p* < 0.05). **g** Confirmation using real time PCR of representative differentially regulated miRNAs between WT and CD47^−^ cells and their EVs (Data S1C). **h** WT cells were transfected with a CD47 guide CRISPR/Cas9and grown in complete medium for 24 h. Transfected cells were transferred into HITES serum free medium for 24 h. EVs were isolated using the Exo-quick Kit, and miRNA levels of miR-31, miR-107 and miR-103 was analyzed using real-time PCR. Significance is indicated for *P*-values ≤ 0.05 for comparisons of Sample and Columns (*) or Sample, Columns and Interaction (**) as detailed in the Statistical Analysis method section. **i** CD47^−^ cells were transfected for 48 h with control or CD47 expression plasmids. Total RNA from cells and EVs was analyzed for expression of the indicated miRNAs. The level was normalized to EVs extracted from WT using U6 as control. Significance was determined by two-sample t-test assuming equal variances (*P*-value ≤ 0.05). **j** WT and CD47^−^ T Cells were transferred into serum free HITES medium for 24 h. EVs were isolated using ultracentrifugation basic protocol 1 and **k** size exclusion chromatography purification using Exo-guidance systems, and real time PCR was performed. **l** RNA was extracted from CD3^+^ T cells isolated from WT and *Cd47*^*−/−*^ mice, and miRNA level was analyzed using real time PCR. Significance is indicated for *P*-values ≤ 0.05 with two-sample t-test: assuming equal variances

To examine the role of CD47 in regulating miRNA levels in cells and EVs, WT and CD47^−^ Jurkat T cells were cultured in an EV-free defined medium (Kaur et al. 2011), and RNAs from the cells and released EVs were subjected to miRNA microarray analysis. Supervised hierarchical clustering of differentially expressed miRNAs in WT and CD47^−^ EVs (Fig. 1a) or in WT and CD47^−^ cells (Fig. 1b) showed limited correlations between levels of CD47-dependent miRNAs in EVs and their cells of origin. Principle component analysis confirmed divergence between CD47-dependent miRNAs in cells versus EVs, cells (Fig. 1c, Data S1, S1A). This divergence is consistent with the reported divergence in global EV versus cell miRNA compositions for Jurkat and two additional T cell lines (Mittelbrunn et al. 2011).

miRNA sequencing was used to confirm the microarray data and more comprehensively identify CD47-dependent miRNAs in cells and in their released EVs. A total of 1194 miRNAs differed significantly between WT and CD47^−^ cells, and 177 miRNAs differed significantly between WT and CD47^−^ EVs (*p* < 0.05, Fig. 1d). Of the latter miRNAs, 113 were CD47-dependent in both cells and EVs. Of these, 28% showed parallel CD47-dependent up- or down-regulation in cells and EVs, which suggests that their levels or maturation in the cells rather than their packaging into EVs is CD47-dependent (Fig. 1d and Data S3). However, most of the miRNAs (72%) exhibited opposing CD47-dependent enrichment in cells versus EVs, which suggested that CD47 actively regulates the packaging of these miRNAs into EVs. The remaining 64 CD47-dependent miRNAs were CD47-dependent only in EVs, and 70% of these were significantly more abundant in the CD47^−^ EVs, indicating that CD47 limits their export in EVs independent of their cellular levels. The global miRNAseq data also indicated that more precursor miRNAs than mature miRNAs exhibited shared CD47-dependence between EVs and cells or uniquely differed in WT versus CD47^−^ EVs (Fig. 1e, f, Data S2A). Therefore, CD47 may preferentially regulate trafficking of precursor rather than mature miRNAs into EVs.

The differential enriched miRNAs in CD47^−^ versus WT EVs or cells included several let7 family members, miR-185, miR-16, miR-103, miR-107, miR-138 miR-16–1, miR-16–2, miR-191, miR-192, miR-193, miR-24, miR-26, miR-27, miR-29 and miR-320a (Data S1C). Real time PCR analysis confirmed that only 4 of the 13 CD47-dependent miRNAs in EVs exhibited a parallel CD47-dependence in cells (black arrows in Fig. 1g). Five members of the miR-320 family were significantly elevated in EVs released by CD47^−^ relative to EVs from WT Jurkat T cells. We previously reported enrichment of miR-320a in CD47^+^ EVs relative to CD63^+^ or MHCI^+^ EVs released from WT Jurkat cells (Kaur et al. 2018a). Consistent with the microarray data for miR-320a, miR-320a-5p was 5.3-fold higher in EVs from CD47^−^ cells compared to EVs from WT cells (Data S2A). Three predicted mRNA targets of miR-320a-5p were among those we previously reported to be differentially regulated in endothelial cells treated with EVs derived from WT versus CD47^−^ Jurkat cells (Data S2B). Analysis of predicted targets for 22 additional top-ranked CD47-dependent miRNAs suggest that these miRNAs primarily contribute to the observed differential effects of WT versus CD47^−^ EVs on mRNA levels in treated endothelial cells (Data S2B).

To confirm that these differences in miRNA levels are CD47-specific and not related to potential secondary mutations in the JinB8 somatic mutant (Reinhold et al. 1999), a CD47 guide was used with CRISPR/Cas9 to knock down CD47 in WT cells, which led to increased levels of miR-107, and miR-31 miRNAs in EVs as compared to mock-treated control cells (Fig. 1h). Real time PCR further confirmed that let-7a-3 and 7f2 have higher levels in CD47^−^ EVs than WT EVs, but only let-7a-3 miRNA was elevated in the absence of CD47 (Data S1C and Figure S2D). Cellular levels of let-7a-3 and miR-107 but not miR-31 were also increased after disruption of CD47 (Fig. 1h). Conversely, transient re-expression of CD47 in CD47^−^ cells increased the levels of miR-103 and miR-107 in the cells but significantly reduced their levels in EVs (Fig. 1I). Levels of miR-31 were not altered in cells. Further analysis of cells selected for stable re-expression of CD47 showed significantly decreased levels of miR-103, miR-107, miR-31, miR-125b, miR-34a and miR-138 (Figure S2E,F, Data S4A).

Differential levels of representative miRNAs were confirmed using EVs isolated by ultracentrifugation (Fig. 1j) or Exo-spin™ size exclusion purification methods and analyzed via real time PCR (Fig. 1k) to compare independence of EV extraction protocols. miR-31, miR-103, and miR-107 were consistently elevated in CD47^−^ versus WT EVs, but less so in the corresponding cells.

CD47-dependence for representative miRNAs was confirmed using primary T cells from WT and *Cd47*^*−/−*^ mice (Lindberg et al. 1996). Levels of miR-31 was significantly elevated in *Cd47*^*−/−*^ CD3^+^ T cells, and miR-103, and miR-107 levels trended higher compared to WT (Fig. 1l).

### CD47 and ubiquilin-1 associate with nuclear pore transport proteins in T cells

**Fig. 2:**
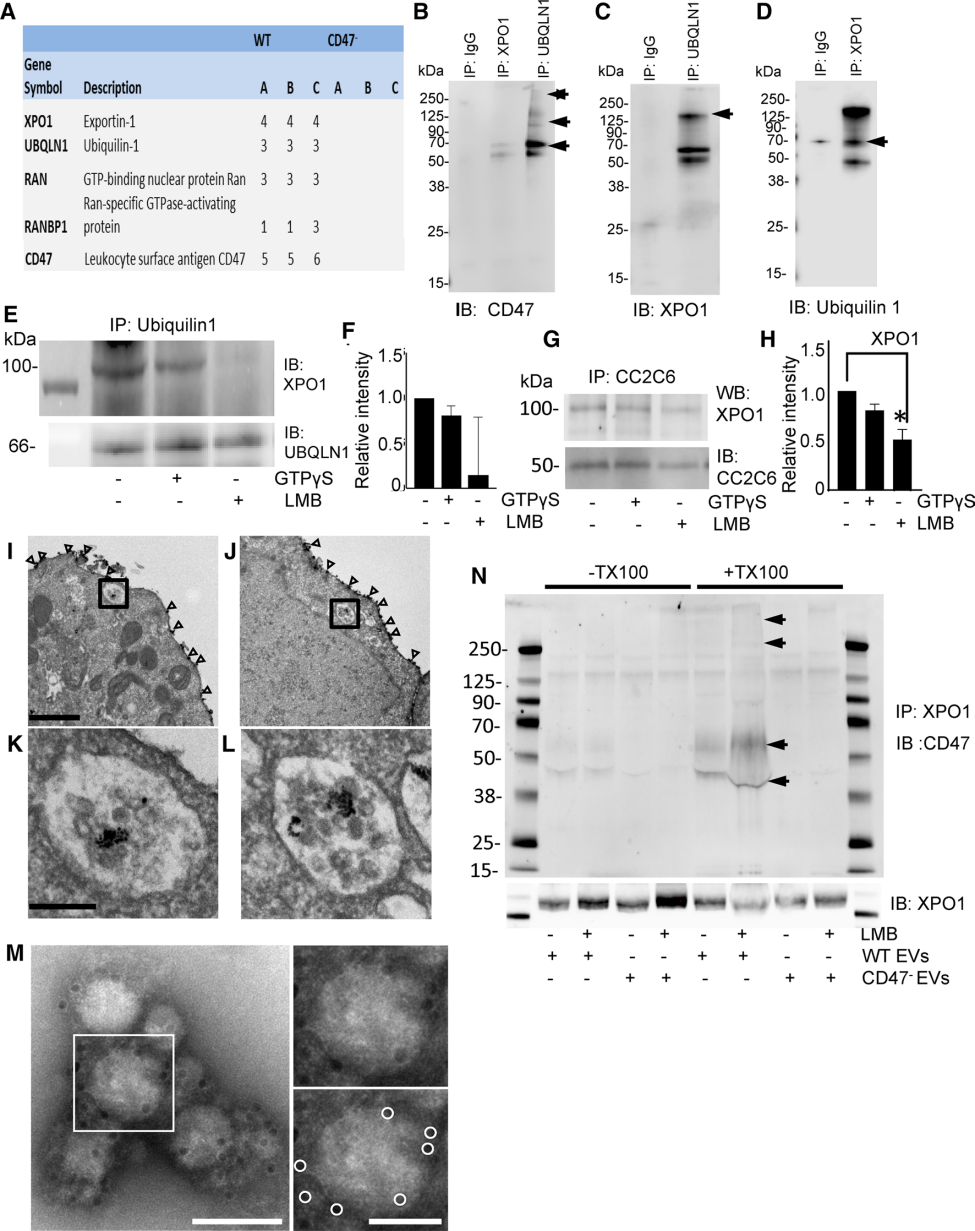
CD47 localization in EVs and interactions with the Ran/exportin-1 complex. **a** Cell lysates from WT and CD47^−^ Jurkat cells were immunoprecipitated with streptavidin beads using a biotin-conjugated CD47 antibody (CC2C6). Proteins eluted from the beads were digested and subjected to LC–MS analysis to identify CD47-interacting proteins. A is the number of unique peptides, those peptides with unique sequence that are only found in the given protein. B is the number of total peptides, which is those with unique sequence that are found in the given protein but may also be found in other proteins. C is the number of peptide spectral matches, which is the total number of MS/MS spectra representing peptides from the given protein, including redundant spectra for the same peptide sequence. **b–d** The interactions identified by LC–MS were validated using immunoprecipitation of CD47, ubiqulin-1 and exportin-1. **e** Representative blot showing effects of LMB and GTPγS treatment on immunoprecipitation of exportin-1 with ubiquilin-1 in Jurkat whole cell lysates **f** Quantitative analysis of 3 replicate experiments with exportin-1 protein density normalized to ubiquilin-1. **g, h** Representative exportin-1 immunoblot and quantification of three replicate experiments (H) showing effects of LMB and GTPγS treatment on the association of exportin-1 with CD47 immunoprecipitated using CC2C6, *p* = .033 (*), **i–l** Electron micrographs for immunogold labeling to localize CD47 in vehicle treated **i, k** and LMB treated (J.L) Jurkat T cells. Open arrowheads indicate CD47 in the plasma membrane. Boxed areas in I and J, enlarged in K and L, respectively, show intracellular labeling for CD47 localized to MVBs. Scale bar for I-J indicates 1 μm; scale bars for K-L indicate 200 nm. **m** CD47 immunogold labeled and negative stained electron micrograph of EVs released by WT T cells and isolated using Exo-spin. Boxed area around one EV is shown enlarged to the right (top), with immunogold particles circled in white below. Scale bars indicate 100 nm (left), and 50 nm (right). **n** Immunoprecipitation of exportin-1 was performed using lysates (± Triton-X100) of EVs derived from WT and CD47^−^ cells or cells treated with 20 nM LMB. Western blotting was performed to detect CD47 (glycosylated isoforms indicated by arrows) and exportin-1

To identify CD47 interactions that could mediate its apparent regulation of RNA trafficking, we analyzed tryptic peptides from biotin-conjugated CD47 antibody pulldowns in detergent lysates of WT and CD47^−^ T cells using HPLC/mass spectrometry (Fig. 2a). Multiple peptides from CD47 and its known cytoplasmic binding partner ubiquilin-1 (encoded by *UBQLN1*) (Wu et al. 1999; N'Diaye and Brown 2003) were identified in WT immunoprecipitates that were absent in immunoprecipitates from the CD47^−^ cells. Ubiquilin-2 is also reported to bind to CD47 (Wu et al. 1999) but was not detected. UBQLN1 mRNA levels determined by RNAseq were similar in the WT and CD47^−^ cells, whereas UBQLN2 mRNA was 5 to eightfold lower, which could account for its absence in the immunoprecipitates.

None of the RNA-binding proteins previously implicated in RNA trafficking to EVs were found (Mittelbrunn et al. 2011; Villarroya-Beltri et al. 2013; Zietzer et al. 2020), but the known RNA trafficking protein exportin-1 and several proteins involved in regulating its nuclear pore transport function including Ran, and RanBP1 (Kohler and Hurt 2007; Castanotto et al. 2009; Martinez et al. 2017) were uniquely pulled down from WT cells. Two additional regulators of exportin-1 function, RanGAP1 and RCC1, were also identified in the pulldown from WT cells, but fewer peptides from these proteins were also detected in the pulldown from CD47^−^ T cells (ftp://MSV000086969@massive.ucsd.edu). Comparison with a previous proteomic analysis of proteins from Hela cells that bound to exportin-1 in GTP-dependent manner (Kirli et al. 2015) revealed three 3 (7.0%) of 43 known nuclear pore transport-related proteins and 25 (3.0%) of the 831 Ran-GTP-dependent exportin-1 cargo proteins identified in Hela cells that were also present in the CD47 pulldown from WT T cells but not from CD47^−^ cells (Data S7). Only 1 (0.4%) of 239 proteins identified as low abundance cargo in HeLa cells was identified by anti-CD47 pulldown, which we take as an upper limit estimate for the false discovery rate in this comparison. Remarkably, ubiquilin-1 was identified by Kirli et al. as an exportin-1-associated protein of unknown function in Hela cells, and ubiquilin-1 binding to immobilized exportin-1 was increased 160-fold in the presence of Ran-GTP (Kirli et al. 2015) (Table 1). Therefore, ubiquilin-1 interacts in a GTP-regulated manner with the exportin-1 complex, suggesting its function as an adapter to mediate the observed association of multiple known exportin-1 cargo proteins with CD47.

**Table 1:** Known RanGTP-dependent exportin-1 binding proteins that associate specifically with CD47 in T cells

Gene	Protein name	CD47 ^−^ peptides	CD47 ^+^ peptides	RanGTP fold Stimulation*	Fold enrichment from input*	Function*
*EIF2S2*	Eukaryotic translation initiation factor 2 subunit 2	0	3	34,000	110	Cargo A
*EIF5B*	Eukaryotic translation initiation factor 5B	0	2	10,000	28.0	Cargo A
*AP1B1*	AP-1 complex subunit beta-1	0	1	12,000	15.0	Cargo A
*AP1G1*	AP-1 complex subunit gamma-1	0	2	4900	10.0	Cargo A
*GNB2*	Guanine nucleotide-binding protein-β2	0	1	3400	14.0	Cargo A
*UBA5*	Ubiquitin-like modifier-activating enzyme 5	0	2	22,000	18.0	Cargo A
*FAM160B1*	Protein FAM160B1	0	1	970	10.0	Cargo A
*GORASP2*	Golgi reassembly-stacking protein 2 (GRS2)	0	3	22,000	16.0	Cargo A
*RPS23*	40S ribosomal protein S23	0	1	1000	5.1	Cargo A
*RTN4*	Reticulon-4 (Foocen, Nogo protein)	0	2	1700	2500	Cargo A
*SEPT2*	Septin-2 (NEDD-5)	0	7	11,000	30.0	Cargo A
*CEP170*	Centrosomal protein of 170 kDa	0	1	2200	14.0	Cargo A
*CSNK2A1/3*	Casein kinase II subunit alpha	0	2	8600	5.5	Cargo A
*PDAP1*	28 kDa heat- and acid-stable phosphoprotein	0	2	1300	24.0	Cargo A
*SKIV2L2*	Superkiller viralicidic activity 2-like 2	0	2	640	4.5	Cargo A
*ARHGEF1*	Rho guanine nucleotide exchange factor 1	0	3	970	2.2	Cargo B
*ETFB*	Electron transfer flavoprotein subunit beta	0	1	2500	2.6	Cargo B
*GSK3B*	Glycogen synthase kinase-3 beta	0	2	1000	2.5	Cargo B
*OCIAD1*	OCIA domain-containing protein 1	0	3	110	300.0	Cargo B
*TUBA1B*	Tubulin alpha-1B chain	0	18	3700	3.0	Cargo B
*UBR4*	E3 ubiquitin-protein ligase UBR4 protein 1	0	3	780	2.1	Cargo B
*WDR61*	WD repeat-containing protein 61	0	2	4000	3.3	Cargo B
*WDR82*	WD repeat-containing protein 82	0	1	550	3.3	Cargo B
*YWHAG*	14–3-3 protein gamma	0	2	2100	2.1	Cargo B
*TTC1*	Tetratricopeptide repeat protein 1	0	2	350	5.3	Cargo B
*VAPA*	VAMP-associated protein A)	0	7	9.1	26.0	LA Cargo
*NUP205*	Nuclear pore complex protein Nup205	0	2	130	0.57	NUP
*RANBP1*	Ran-binding protein 1 (RanBP1)	0	1	13,000	1.1	CRM1 cofactor
*IPO5*	Importin-5 (RanBP5)	0	7	42	0.0	NTR
*UBQLN1*	Ubiquilin-1 (PLIC-1)	0	3	160	0.6	ambiguous

The number of peptides identified by LC/MS analysis is presented for each protein in the CD47 antibody bead pulldowns from detergent solubilized lysates of WT (CD47^+^) and CD47^−^ T cells. Entries in the table represent the intersection between all identified CD47-specific proteins in Jurkat T cells and the RanGTP-dependent exportin-1-binding proteins identified in Hela cell extracts by Kirli et al. (Kirli et al. 2015). *RanGTP fold stimulation, fold enrichment, and function data are from (Kirli et al. 2015): Cargo A = RanGTP stimulation ≥ 500 with enrichment ≥ 4 or enrichment ≥ 100 with RanGTP stimulation 2–499-fold and mole fraction in exportin-1/RanGTP pulldown > 50 ppm, Cargo B = RanGTP stimulation > twofold and enrichment > twofold and mole fraction in exportin-1/RanGTP pulldown > 50 ppm, LA Cargo = Low abundance cargo: mole fraction in exportin-1/RanGTP pulldown > 1 ppm, NUP = nuclear pore protein, NTR = nuclear transport related

Binding of ubiquilin-1 to the C-terminal cytoplasmic tail of CD47 was first established using a yeast two-hybrid screen (Frazier et al. 1999; Wu et al. 1999). Ubiquilin-1 serves as an adapter to mediate CD47-dependent cytoskeletal regulation and heterotrimeric G protein signaling (N'Diaye and Brown 2003). We confirmed CD47 interactions with ubiqulin-1 and exportin-1 using IP-western blotting (Fig. 2b–d). CD47 was strongly enriched in a ubiquilin-1 IP as expected, whereas the exportin-1 IP enriched CD47 to a lesser degree (Fig. 2b). Conversely, exportin-1 was strongly enriched in a ubiquilin-1 IP (Fig. 2c), suggesting that ubiquilin-1 mediates an indirect interaction between CD47 and the exportin-1 complex. Consistent with this model, immunoprecipitation of exportin-1 efficiently pulled down ubiquilin-1 (Fig. 2d).

Exportin-1 recruits cargo when its regulator Ran is in the GTP-bound active state (Monecke et al. 2014). RanBP1, which binds to Ran and stimulates GTP hydrolysis, and the regulatory binding protein RanBP3 regulates nuclear-cytoplasmic shuttling of proteins and RNA via exportin-1 (Plafker and Macara 2000). Co-immunoprecipitation was used to further examine effects of the nonhydrolyzable GTP analog guanosine 5′-[γ-thio]-triphosphate (GTPγS) and leptomycin-B (LMB), which specifically alkylates exportin-1 at Cys^528^ and thereby inhibits its nuclear export function (Kudo et al. 1999), on the interactions of CD47 and ubiquilin-1 with the exportin-1 complex (Fig. 2e–h and Figure S3A-D). Pretreatment of WT Jurkat T cells with LMB significantly inhibited the association of exportin-1 with ubiquilin-1, whereas treatment of cell lysates for 15 min with GTPγS on ice before immunoprecipitation had minimal effects on the same interaction (Fig. 2e, f). Immunoprecipitation of cytosolic WT lysates using anti-CD47 confirmed interactions with RanGAP1, RanBP1, RanBP3, and exportin-1 (Figure S3A-D). GTPγS treatment increased CD47 interaction with RanBP1 in some experiments, but in aggregate this did not achieve significance (Figure S3A,C). RanGAP1 and RanBP3 also did not show significant GTP-dependence for their interactions with CD47 (Figure S3B-D). LMB also failed to significantly alter the interactions of CD47 with RanBP1 and RanBP3 (Figure S3C-D). On the other hand, LMB treatment significantly reduced the association of exportin-1 with CD47 in cytoplasmic extracts immunoprecipitated using the CD47 antibody CC2C6, which recognizes the mature pyro-Glu modified form of CD47 after passage through the endoplasmic reticulum (Logtenberg et al. 2019) (Fig. 2g, h). Interpretation of the lack of significant GTPγS effects in the CD47 and ubiquilin-1 IPs may be limited by its additional effects on heterotrimeric G protein interactions with CD47 (Frazier et al. 1999) and ubiquilin-1 that could mask GTP-dependent Ran binding.

### LMB treatment increases release of CD47 and exportin-1 into EVs

Exportin-1 was identified as an abundant protein in EVs released by prostate cancer cells (Duijvesz et al. 2013), and we observed increased exportin-1 protein in EVs derived from CD47^−^ T cells as compared to EVs from WT cells using western blot analysis of the 20–40% density gradient fraction but not in the 40–60% gradient fraction (Figure S3E). Exportin-1 (green) and actin (red) were visualized by confocal imaging of WT Jurkat T cells immobilized on poly-lysine before and after treatment with LMB (Figure S3F). As shown in the representative images, treatment of cells with LMB for 1 h resulted in the release of exportin-1^+^ vesicular material in 19% of cells. Less than 6% of untreated cells showed similar release of exportin-1^+^ vesicles.

Confocal microscopy analysis of WT and CD47^−^ cells treated with LMB for one hour showed less retention of exportin-1 in the nucleus for CD47^−^ cells and more accumulation in the cytoplasm (Figure S3G,H). LMB treatment also decreased the level of CD47 remaining on the cell surface as assessed by flow cytometry using two different antibodies (Figure S3I). Similarly, LMB treatment decreased cell surface staining of CD47 assessed by confocal microscopy (Figure S3J). CD47 is a highly modified protein with isoforms that bear N-glycosylation (50–60 kDa) and O-linked glycosaminoglycan modification (90–200 kDa) (Kaur et al. 2011). Direct western blotting indicated increased glycosylated isoforms of CD47 in released EVs from LMB-treated Jurkat T cells (Figure S3K).

Release of CD47 in EVs may occur directly from the plasma membrane or via the MVB pathway. Immunogold labeling identified CD47 in a subset of vesicles within MVBs visualized by electron microscopy in Jurkat T cells (Fig. 2i–l, Figure S4A,B,L). CD47^+^ EVs were identified by staining with anti-CD47-immunogold, and their size was evaluated with electron microscopy (Fig. 2m). CD47 immunogold particles (white dots) were enriched in vesicles ~ 75 nm in diameter. Treatment with LMB did not alter the number of MVBs or the abundance of CD47-expressing vesicles therein (Fig. 2j, l Figure S4C-K). Therefore, some release of CD47-expressing EVs occurs via the MVB pathway, but exportin-1 probably does not regulate the release of CD47-expressing EVs at the level of MVB biogenesis. Loss of cell surface CD47 following LMB treatment (Figure S4G-J) is consistent with exportin-1 regulating release of CD47-expressing EVs from the plasma membrane, but the present data does not exclude exportin-1 regulation of release of CD47-expressing EVs via MVBs.

To determine whether CD47 in EVs associates with exportin-1, EVs lysates from untreated and LMB-treated WT and CD47^−^ T cells were subjected to exportin-1 immunoprecipitation. The EVs lysates were heated with LDS buffer or in combination with TX-100, and western blotting was performed using exportin-1 and CD47 antibodies. Consistent with the release of exportin-1^+^ vesicles in Figure S3E, LMB treatment enhanced the levels of exportin-1 and associated CD47 in EVs (Fig. 2n). Glycosylated CD47 isoforms were more efficiently solubilized in the presence of Triton-X100 and were enhanced on exportin-1 immunoprecipitates from EVs following LMB treatment. Notably, LMB treatment of CD47^−^ cells induced a greater release of exportin-1 into EVs compared to that released into EVs from WT cells. In contrast to CD47, exportin-1 was solubilized more efficiently in the absence of Triton-X100 and was more abundant in EVs from LMB-treated cells. The LMB-induced increase in exportin-1 in EVs was confirmed in a second experiment that also examined regulators of its function (Figure S4M). Ubiquilin-1 was associated with exportin-1 in WT and CD47^−^ EVs and not inhibited by LMB treatment. RanGAP1 and RanBP3 were also associated with exportin-1 in WT and CD47^−^ EVs and not affected by LMB. Association of RanBP1 with exportin-1 was more evident in CD47^−^ EVs when treated with LMB.

### CD47-dependence of exportin-1-dependent miRNAs

**Fig. 3:**
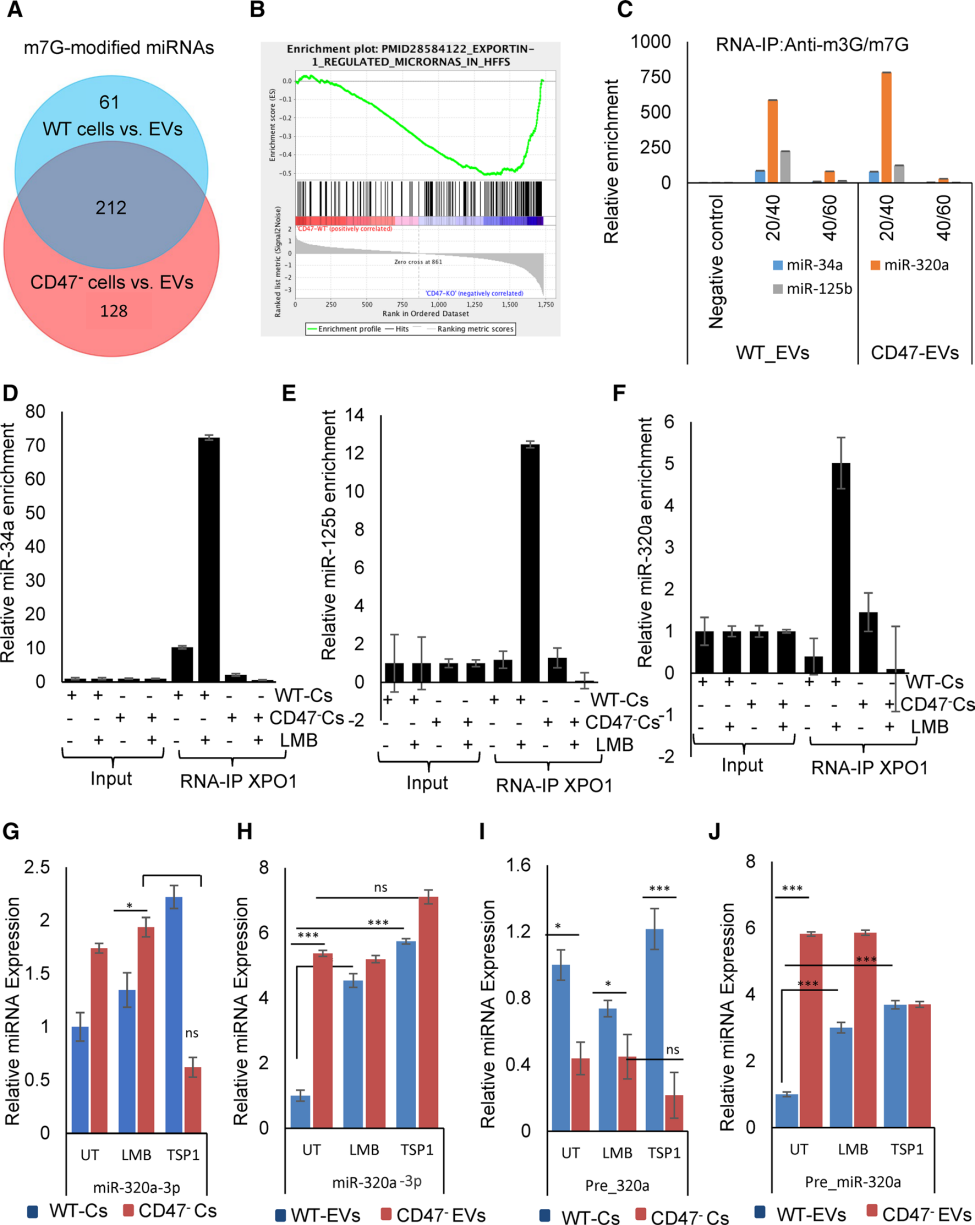
CD47 regulates m^7^G-modified miRNAs in EVs released by Jurkat T cells and their association with exportin-1. **a** Analysis of known m^7^G-modified miRNAs that were differentially expressed in WT (blue) or CD47^−^ (red) EVs vs cells (*p* < 0.05) (from Data S2C, Fig S4N,O). **b** Gene set enrichment analysis (GSEA) of exportin-1-dependent miRNAs in EVs released from WT versus CD47^−^ cells. **c** EVs released by WT and CD47^−^ cells were isolated by density gradient centrifugation for 16 h, lysed, and levels of the indicated miRNAs was analyzed. RNA–IP was performed from the same lysates using m^3^G/m^7^G-cap antibody, and miRNAs were quantified using real-time PCR and normalized to input. **d–f** CD47-dependent association of known m^7^G-cap-dependent pre-miRNAs with exportin-1. Association of the indicated pre-miRNAs with exportin-1 was quantified using RNA-immunoprecipitation with anti-exportin-1 from WT and CD47^−^ T cells followed by qPCR analysis. Enrichments were calculated after normalizing all input values to one. **g–j** Effects of TSP1 and inhibiting exportin-1 with LMB on miRNA maturation. miRNA levels of miR-320a-3p and pre-miR-320a (Qiagen) in untreated and TSP1 treated WT and CD47^−^ T cells and released EVs purified using Exo-spin™

Previous studies have identified specific roles for exportin-1 in the biogenesis of miRNAs that are modified by m^7^G or 2,2,7-trimethylguanosine (TMG) (Castanotto et al. 2009; Martinez et al. 2017; Sexton et al. 2019). Of the 177 CD47-dependent miRNAs in EVs, 73 were previously identified as bearing m^7^G modifications (Pandolfini et al. 2019). Less overlap (14%) was found with senescence-associated miRNAs identified as exportin-1-dependent based on TMG modification (Martinez et al. 2017), while 58% overlapped with m^7^G-modified miRNAs identified in two previous studies (Data S3) (Castanotto et al. 2009; Sexton et al. 2019). Global analysis of the miRNA-seq data (Data S2C) identified 273 miRNAs that differed in WT cells versus EVs with *p* < 0.05 (Figure S4N and 340 that differed in CD47^−^ cells versus EVs (Figure S4O). Of these, 212 were common between WT and CD47^−^ cells, but more m^7^G-modified miRNAs differed between CD47^−^ EVs and cells than differed between WT EVs and cells (Fig. 3a).

Let7a-3p (Fig. 1h) and miR-320a-5p were among the CD47-dependent miRNAs that were previously identified as bearing m^7^G modifications (Pandolfini et al. 2019). The biogenesis of miR-320a is independent of conventional miRNA biogenesis involving Drosha endonuclease cleavage and exportin-5-dependent transport to the cytoplasm (Chong et al. 2010; Xie et al. 2013). Precursor miR-320a (pre-miRNA) contains a m^7^G-modification and is exported from the nucleus by the exportin-1 transporter complex (Xie et al. 2013). Gene set enrichment analysis of the microarray data confirmed that exportin-1-dependent miRNAs are enriched in EVs derived from CD47^−^ cells (Fig. 3b). EVs from WT and CD47^−^ T cells were isolated by density gradient centrifugation (Figure S5A) and their miRNAs contents analyzed. miR-320a and two control microRNAs that were not CD47-dependent (miR-34a and miR-125b) were enriched in the 20–40% as compared to 40–60% fraction (Figure S5B), consistent with the known density of EVs (Shurtleff et al. 2016). As expected, EVs derived from CD47^−^ T cells expressed higher levels of miR-320a but not of miR125b and miR34a (Figure S5B). Consistent with these data, RNA immunoprecipitation using a m^3^G-cap/m^7^G-cap antibody enriched miR-320a to a greater degree relative to input than miR-125b or miRNA-34a (Fig. 3c). Therefore, enrichment of these miRNAs in EVs correlates with their degree of m^7^G-modification. However, the extent of m^7^G-modification of miR-320a and miR-34a did not differ significantly between EVs from CD47^−^ versus WT cells (Fig. 3c). Therefore, CD47 appears to limit the level of secreted or released m^7^G-modified miRNAs in EVs rather than their efficiency of modification. Gene set enrichment analysis of the microarray data further confirmed that exportin-1-dependent miRNAs are enriched in EVs derived from CD47^−^ human T cells (Fig. 3b and Data S5).

Similar CD47-dependent enrichment of m^7^G-modified miRNAs was observed in EVs isolated from WT and *Cd47*^*−/−*^ mouse plasma. We identified 26 differentially expressed miRNAs (Data S4B panel A). Of these, 6 miRNAs are known to be exportin-1-dependent (Data S4 Panel B), and three (mmu-miR-130b, mmu-miR-376c and mmu-miR-33) were previously identified as m^7^G dependent (Sexton et al. 2019). Consistent with the human and mouse T cell data, most of these miRNAs were higher in EVs from *Cd47*^*−/−*^ plasma relative to WT plasma. These data further confirm that CD47 regulation of m^7^G-modified miRNA secretion is not limited to humans.

### LMB increases CD47-dependent association of miRNAs with exportin-1

The data in Fig. 3 indicated greater m^7^G-modification of miR320a and higher miR320a copy number from CD47^−^ EVs as compared to WT (Figure S5C,D), but levels in the cells were not increased (Figure S5D). hsa-miR34a and hsa-miR125b are also known to associate with exportin-1 (Xie et al. 2013; Martinez et al. 2017), and cellular levels were 2- to fourfold higher relative to U6 controls in CD47^−^ versus WT cells (Figure S5E). LMB treatment differentially altered the association of these miRNAs with exportin-1 in a CD47-dependent manner. RNA-immunoprecipitation was performed using an exportin-1 antibody from untreated CD47^−^ and WT T cells and cells treated for 4 h with LMB. LMB treatment strongly enhanced the association of hsa-miR34a, hsa-miR125b and hsa-miR320a-3p with exportin-1 in WT T cells but not in CD47^−^ T cells (Fig. 3d–f). Examination of expression levels before input normalization demonstrated that LMB treatment also increased the level of hsa-miR34a, has-miR125b and has-miR320a-3p in the input samples prepared from CD47^−^ cells but had weaker effects on the input levels from WT cells (Figure S5F-H).

One potential mechanism to account for the preceding data is that CD47 regulates m^7^G-modified miRNA maturation. Alternatively, CD47 could modulate loading these specific microRNAs into EVs for secretion. Because the miR-320 family is known to be m^7^G modified, we confirmed the expression of mature and precursor miR-320a in WT and CD47^−^ T cells and EVs released by these cells (Fig. 3g–j). Basally, miR-320a and pre-miR-320a were expressed at higher levels in EVs (Fig. 3h,j) relative to expression in their parental cells (Fig. 3g, i). Consistent with the microarray data, miR-320a-3p and pre-miR-320a were higher basally in EVs from CD47^−^ cells compared to the respective WT EVs (Fig. 3h, j). Cellular miR-320a-3p also showed CD47-dependence (Fig. 3g), but cellular pre-miR-320a was lower in CD47^−^ cells relative to WT cells, suggesting an additional role for CD47 in regulating maturation of miR-320a (Fig. 3i).

Treatment of WT or CD47^−^ cells with LMB did not significantly alter mature miR-320-3p levels (Fig. 3g), whereas LMB treatment elevated miR-320a-3p expression in EVs from WT cells to match that in EVs from CD47^−^ cells (Fig. 3h), suggesting that CD47 negatively regulates miR-320a levels in EVs by a mechanism that requires exportin-1 function. A similar pattern of response to LMB was observed for pre-miR-320a, where cellular levels did not show a significant response to LMB treatment, but LMB selectively increased pre-miR-320a in EVs released from CD47^−^ cells (Fig. 3i, j). This further suggests that the CD47-limited loading of mature and pre-miR-320a into EVs requires exportin-1 function.

Treatment of WT cells with the CD47 signaling ligand TSP1 significantly increased mature miR-320a-3p levels in WT cells and EVs but not in CD47^−^ cells or EVs (Fig. 3g, h). Similar CD47-dependent regulation by TSP1 of pre-miR-320a was seen in EVs but not in cells (Fig. 3i, j). This suggests that TSP1 signaling via CD47 enhances exportin-1-mediated miR-320a cargo packaging into EV.

### CD47 limits EIF4E-dependent capped mRNAs in EVs released by Jurkat T cells

**Fig. 4:**
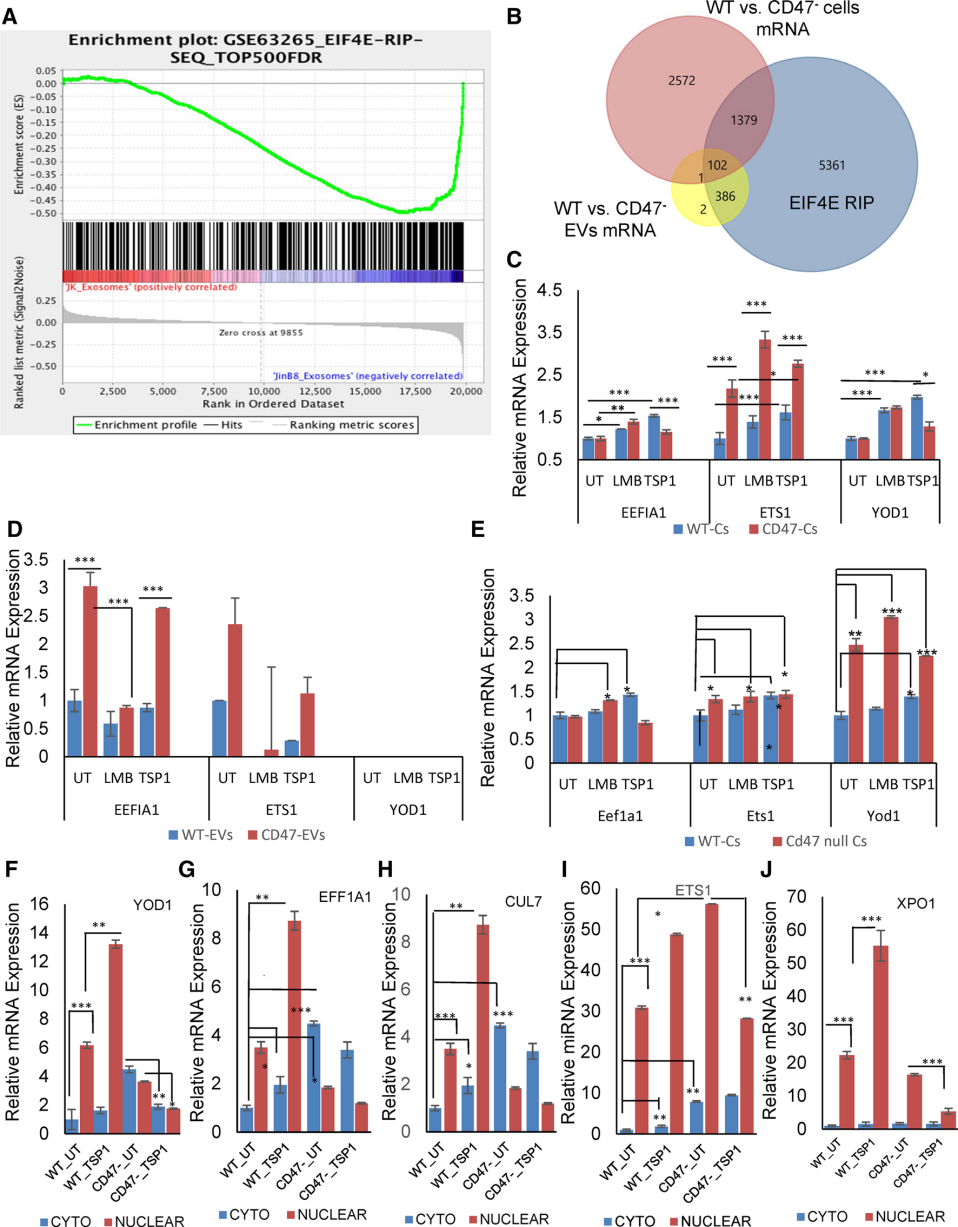
CD47 and TSP1 regulation of m^7^G-cap-dependent mRNA trafficking in human and murine T cells. **a** GSEA analysis shows enrichment of EIF4E-dependent RNAs in EVs released from CD47^−^ versus WT Jurkat T cells. **b** Venn Diagram representing overlap between CD47-dependent mRNAs in cells and EVs with previously reported EIF4E-dependent capped RNAs (Culjkovic-Kraljacic et al. 2016). **c, d** Expression of YOD1, EEFIA1 and ETS1 mRNAs in untreated WT and CD47^−^ Jurkat T cells and cells treated with TSP1 or LMB. EVs were isolated using Exo-spin™ technology. **e** Expression of Yod1, Eefia1 and Ets1 in control, and LMB or TSP1 treated WT and *Cd47*^*−/−*^ mouse T cells using total cellular RNA. **f**–**j** Cytosolic and nuclear mRNA expression of YOD1, EEFIA1, CUL7, ETS1 and XPO1 in untreated WT and CD47^−^ Jurkat T cells and cells treated with 1 μg/ml TSP1. *p* < 0.05 (*), *p* < 0.01 (**), p < 0.001 (***)

To determine whether CD47-dependent regulation of m^7^G-modified RNAs extends to m^7^G-capped mRNAs, we analyzed our previously published microarray data for mRNAs differentially present in EVs released from WT versus CD47^−^ cells using a dataset of cap-dependent mRNAs identified in diffuse B cell lymphoma cells by RNA sequencing of nuclear EIF4E immunoprecipitates (Culjkovic-Kraljacic et al. 2016). Differentially expressed transcripts in CD47^−^ versus WT EVs were highly enriched in EIF4E-dependent mRNAs (Fig. 4a, b). Compared to the cells where 36.5% of the mRNAs are known to be EIF4E-dependent, 99.4% of the CD47-dependent mRNAs in the EVs derived from these cells were EIF4E-dependent (Fig. 4b). Of the CD47-dependent mRNAs in EVs, (94%), including ZBTB40, were significantly elevated in CD47^−^ EVs relative to WT EVs, and 6% were less abundant in CD47^−^ EVs (Data S6). Therefore, enrichment of CD47-dependent m^7^G-capped RNAs is much greater in EVs than the cells from which they are released, and CD47 globally limits their release into EVs. Furthermore, a majority of the CD47-dependent m^7^G-capped RNAs in EVs (79%) did not exhibit CD47-dependence in the cells, indicating that CD47 primarily regulates their trafficking to EVs rather than their cellular expression (Fig. 4b).

TSP1 treatment increased cellular expression of the cap-dependent mRNAs EEFIA1, YOD1 and EST1 in WT but not in CD47^−^ Jurkat T cells (Fig. 4c). LMB treatment significantly enhanced mRNA expression of EEFIA1, EST1 and YOD1, but this increase was independent of CD47 expression. Analysis of EVs released by Jurkat cells receiving the same treatments confirmed that EEFIA1 and ETS1 mRNA levels are significantly higher in CD47^−^ as compared to WT EVs (Fig. 4d). YOD1 was undetectable in WT and CD47^−^ EVs, and correspondingly was not found in our previous microarray analysis of EVs from these cells. TSP1 treatment did not alter the differential expression of EEFIA1 or ETS1 in WT versus CD47^−^ EVs, but the level of ETS1 in EVs from WT cells was slightly inhibited by TSP1 treatment. LMB treatment significantly inhibited the level of EEFIA1 mRNA in CD47^−^ EVs but not in WT EVs. Comparing the cellular and EV data suggests that CD47 limits EEFIA1 mRNA packaging into EVs by a mechanism that requires exportin-1 function.

The same three genes were examined in primary CD3^+^ T cells from WT and *Cd47*^*−/−*^ mice (Fig. 4e). TSP1 treatment increased expression of the cap-dependent mRNAs Eefia1, Yod1 and Est1 in WT but not in *Cd47*^*−/−*^ CD3^+^ T cells.

One potential mechanism by which CD47 could globally regulate cap-dependent mRNA abundance in EVs is by inhibiting EIF4E-mediated export of these mRNAs from the nucleus via the exportin-1/CRM1 pathway (Culjkovic-Kraljacic et al. 2016). Nuclear levels of 5 known m^7^G cap-dependent mRNAs in WT and CD47^−^ T cells did not correlate with their enrichment in CD47^−^ EVs (Fig. 4f–j). In contrast, cytoplasmic levels of these mRNAs were consistently higher in CD47^−^ cells. Notably, treating WT cells with the CD47 ligand TSP1 increased the nuclear levels of the same mRNAs, but this response was lost or in some cases reversed in CD47^−^ nuclear extracts, indicating that the stimulatory activity of TSP1 in WT cells is CD47-dependent. Signaling through other TSP1 receptors (Soto-Pantoja et al. 2015) may mediate its weaker inhibitory activity in CD47^−^ cells. Several of the transcripts showed significant TSP1-induced increases for their cytoplasmic levels in WT cells that were absent or reversed in CD47^−^ cytoplasmic extracts. Therefore, TSP1 has CD47-dependent effects on the nuclear and cytoplasmic levels of cap-dependent transcripts and their release into EVs.

### TSP1 modulates interactions of CD47 with the exportin-1 complex

**Fig. 5:**
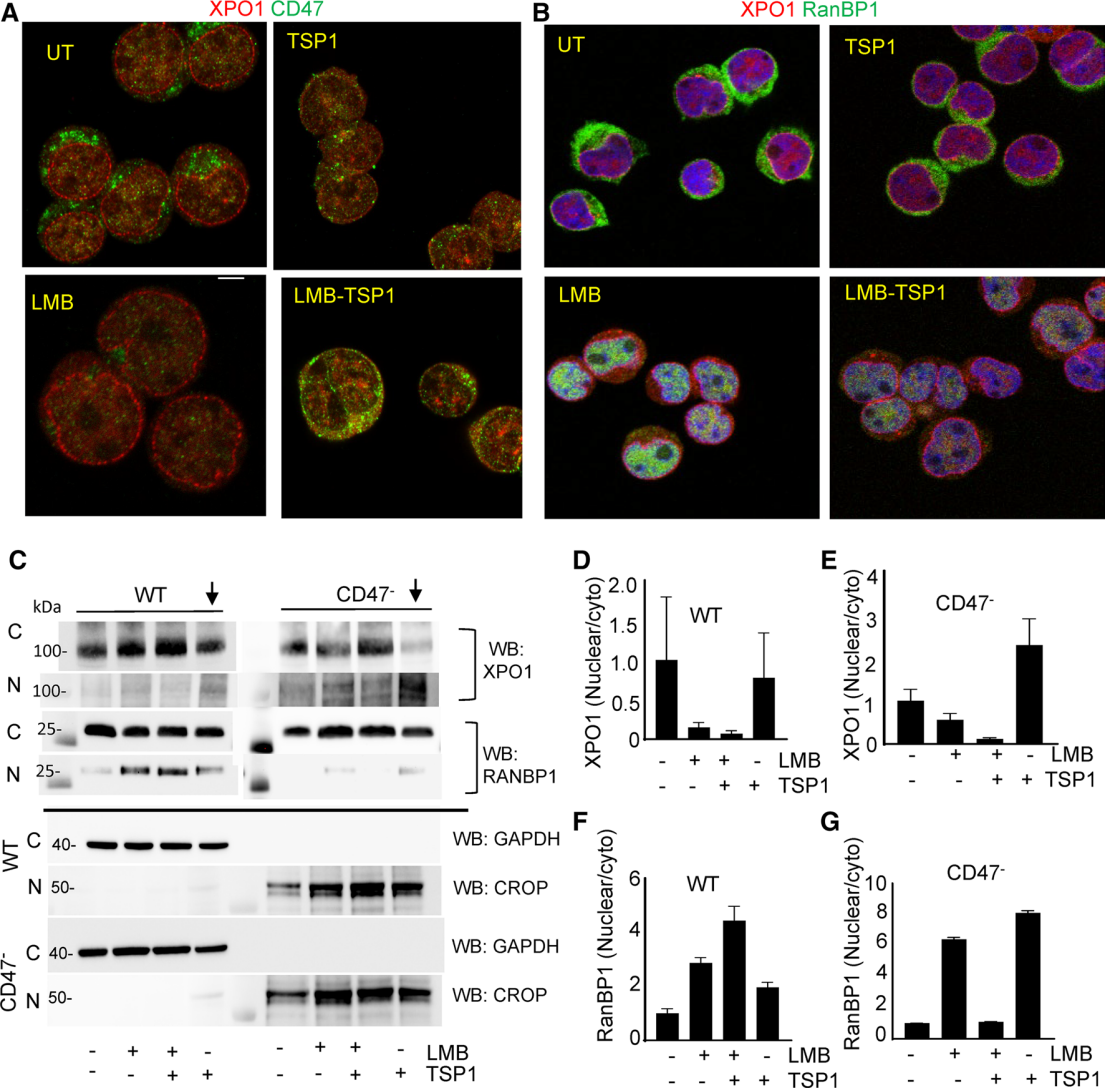
TSP1 regulates the subcellular localization of exportin-1 and RanBP1 in a CD47-dependent manner. **a, b** Confocal microscopic analysis of CD47/exportin-1 and exportin-1/RanBP1 in T cells treated with LMB and TSP1 (1 μg/ml) for 1 h. Scale bar = 5 μm. **c** Western blots with anti-exportin-1 and RanBP1 in cytosolic, nuclear, and EV fractions from untreated WT and CD47^−^ T cells and cells treated with LMB and TSP1. **d**–**g** Quantification of western blots by calculating the ratio of nuclear/cytosolic was determined by normalizing to immunoblots for GAPDH or CROP loading controls, respectively (n = 2)

Confocal analysis of WT cells treated with TSP1 revealed a decrease in cytoplasmic CD47 staining and staining of exportin-1 at the nuclear envelope and increased staining proximal to the plasma membrane (Fig. 5a). LMB treatment decreased exportin-1 staining at the nuclear envelope. However, co-treatment with TSP1 and LMB led to enhanced CD47 in the cytoplasm as compared to single treatment of LMB and a loss of exportin-1 on the nuclear envelope (Fig. 5a). RanBP1 was mostly cytoplasmic in untreated cells, and TSP1 treatment had minimal effects on RanBP1 localization based on similar Lookup Analysis profiles. LMB treatment redistributed RanBP1 into the nucleus as expected. However, TSP1 treatment in the presence or absence of LMB resulted in decreased nuclear RanBP1 staining (Fig. 5b, Figure S6A).

Western blotting of cytoplasmic and nuclear fractions from WT and CD47^−^ cells demonstrated that TSP1 treatment alone had a minimal effect on the overall nuclear/cytoplasmic distribution of exportin-1 in WT cells. TSP1 treatment moderately enhanced the nuclear fraction of exportin-1 in CD47^−^ cells, but this was associated with a decrease in cytoplasmic exportin-1 (Fig. 5c upper panels, d, e). LMB treatment alone decreased the nuclear/cytoplasmic fraction for exportin-1 in WT and CD47^−^ cells. By further increasing cytoplasmic exportin-1, combining LMB with TSP1 further decreased the nuclear/cytoplasmic ratio relative to LMB alone in WT and CD47^−^ cells. Therefore, the subcellular localization of exportin-1 is not a major target of CD47-dependent TSP1 signaling, but TSP1 may increase nuclear exportin-1 through a receptor other than CD47.

A role for CD47 was more evident in the nuclear/cytoplasmic partitioning of RanBP1 (Fig. 5c,f,g). Consistent with the confocal imaging, LMB treatment alone or with TSP1 increased the nuclear/cytoplasmic fraction of RanBP1 in WT cells, and TSP1 had a weak stimulatory effect. In contrast, CD47^−^ cells showed minimal basal RanBP1 in the nucleus, and TSP1 prevented the increase stimulated by LMB treatment (Fig. 5c Middle panel, f, g). Consistent with the exportin-1 data, TSP1 effects on RanBP1 localization may be CD47-independent.

To further determine whether apparent changes in the overall cellular levels of exportin-1 and RanBP1 could be explained by their release in EVs induced by LMB or TSP1, we examined conditioned media collected from same experiments and separated larger vesicles (10,000xg pellets) and small EVs (Total EVs, Figure S6B). Western blots performed for exportin-1 and RanBP1 indicated that CD47^−^ cells release more exportin-1 associated with small and larger EVs relative to EVs from WT cells. RanBP1 was much higher on CD47^−^ EVs than WT EVs but at similar levels on larger vesicles released from WT and CD47^−^ cells. Exportin-1 expression on large and small EVs from WT cells remained undetectable after treatments with TSP1 or LMB (Figure S6C). To improve the detection limit, we performed immunoprecipitation of exportin-1 and found that CD47^−^EVs contained more RanBP1, but its association with exportin-1 was not significantly CD47-dependent and was not significantly altered by LMB or TSP1 treatment of the cells (Figure S6C,D,E).

## Discussion

The present data provide a molecular basis for the previously reported CD47-dependent regulation of the mRNA composition and functional activities of EVs released by T cells (Kaur et al. 2014b, 2018a). Many of the mRNAs previously identified to be elevated in EVs released by CD47^−^ T cells are m^7^G-capped and thus are EIF4E-mediated cargo of the CRM1/exportin-1 nuclear export complex. The present results show that the CD47-regulated enrichment of m^7^G-modified RNAs extends to miRNAs, which can enable broad regulation of gene expression in cells that take up these EVs. Many of the top-ranked CD47-dependent miRNAs in T cell-derived EVs, including the m^7^G-dependent miR-320a-5p, are known regulators of angiogenesis ((Sun et al. 2017) and additional references in Data S2B) and have predicted or known mRNA targets that were differentially regulated by treatment of endothelial cells with EVs derived from WT versus CD47^−^ T cells (Kaur et al. 2014b). Among these, let-7a-3p, miR-185, and miR-139-5p were previously reported to regulate endothelial angiogenic functions when delivered via EVs (Anene et al. 2018; Lu et al. 2021; Si et al. 2021), Therefore, CD47-dependent packaging of these and other miRNAs into EVs may account for the previously reported CD47-dependent regulation of angiogenic gene expression and signaling in recipient endothelial cells (Kaur et al. 2014b).

**Fig. 6:**
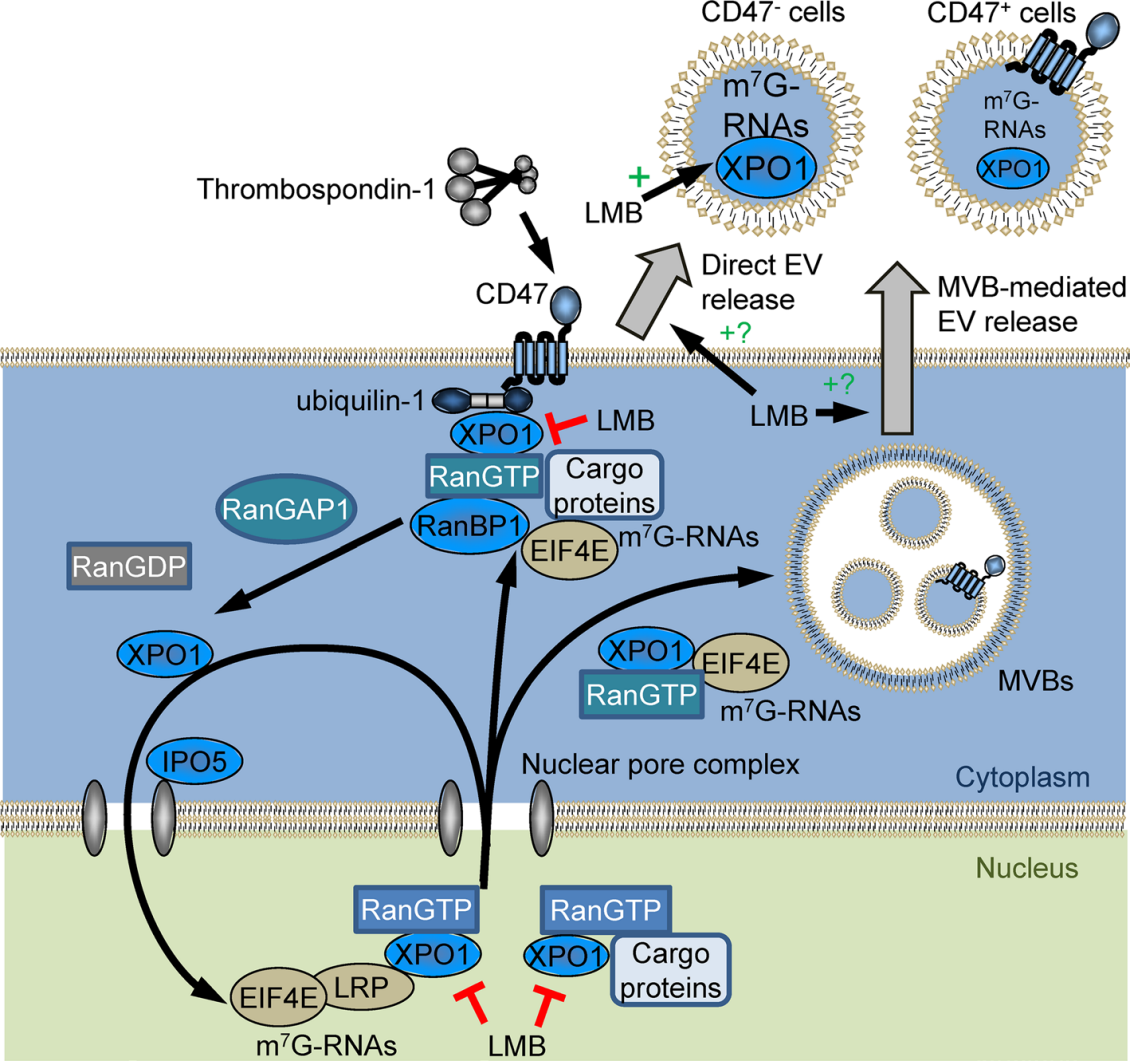
CD47/ubiquilin-1 interact with and regulate the Ran/exportin-1 nuclear transport complex, which mediates trafficking of m^7^G-modified RNAs to the cytoplasm and extracellular vesicles (EVs). CD47 signaling regulates nuclear/cytoplasmic transport of m^7^G-capped RNAs and their abundance in EVs. Leptomycin B (LMB) inactivates exportin-1 (XPO1) by covalent modification, which inhibits the export of cargo proteins and m^7^G-capped RNAs from the nucleus. LMB also inhibits the association of exportin-1 with CD47 and enhances levels of m^7^G-capped RNAs in EVs released from cells. The mechanism by which exportin-1 and CD47 regulate RNAs destined for direct EV release versus release via multivesicular bodies (MVBs) remains to be determined

Furthermore, we demonstrate physical interactions of CD47 and its cytoplasmic signaling adapter ubiquilin-1 with exportin-1 and several regulators of its nuclear export complex. Inhibition of exportin-1 function by the highly specific covalent inhibitor LMB perturbs the CD47-dependent trafficking of m^7^G-capped RNAs between nuclear and cytoplasmic compartments and their export via EVs (Fig. 6). This proposed mechanism is further supported by the previous identification of ubiquilin-1 as a GTP-dependent binding partner of exportin-1 in Hela cells (Kirli et al. 2015). We further establish that the CD47 signaling ligand TSP1 regulates the exportin-1 transport complex and its m^7^G-capped RNA cargo. The regulation of exportin-1 dependent RNAs by CD47 and TSP1 that we identified in human and murine T cells may have a broader physiologic significance because a similar enrichment of m^7^G-cap-dependent miRNAs was found in circulating plasma EVs from *Cd47*^*−/−*^ versus WT mice.

Most of the biochemical data was obtained using a human T cell line mutant that lacks CD47 expression, but the central conclusions were validated using CRISPR/Cas9 to disrupt CD47 in the WT T cell line and in murine CD3^+^ T cells and plasma EVs from mice with germline disruption of the *Cd47* gene. Conversely, transient or stable re-expression of CD47 in the CD47^−^ human T cell line restored WT phenotypes for several exportin-1-dependent miRNA and mRNAs.

An unbiased proteomic analysis identified physical associations of CD47 with several proteins in the exportin-1 nuclear export complex and some of its known cargo proteins. Specific associations between CD47 and exportin-1 and RanBP1 in EVs and between CD47 and exportin-1, RanBP1, RanBP3, RanGAP1 in cells were confirmed by co-immunoprecipitation. Our results further confirm the direct interaction of CD47 with ubiquilin-1 (Wu et al. 1999; N'Diaye and Brown 2003). Mass spectrometry and co-immunoprecipitation identified ubiquilin-1 interactions with CD47 and with exportin-1 in T cells, which is consistent with the previous identification of ubiquilin-1 as a GTP-dependent exportin-1 binding partner of unknown function in Hela cells (Kirli et al. 2015). Co-immunoprecipitation suggests that the interaction between ubiquilin-1 and exportin-1 is stronger than that between CD47 and exportin-1, which is consistent with ubquilin-1 being an adapter that mediates binding of the Ran/exportin-1 complex to CD47. However, further studies are needed to confirm which interactions are direct versus indirect.

Previous studies have identified diverse functions of the five human ubiquilins, including regulating protein stability, DNA damage responses, cytoskeletal organization, and autophagy (Zheng et al. 2020), several of which overlap with known CD47 signaling functions. Although our data and that of (Kirli et al. 2015) suggest that ubiquilin-1 specifically mediates the CD47-exportin-1 interaction, we caution that the low expression of ubiquilin-2 in T cells does not exclude its potential function in other cell types.

In addition to establishing physical interactions, our data provides preliminary evidence supporting molecular mechanisms by which CD47 signaling regulates function of the exportin-1/RanGTP complex. m^7^G-capped RNAs are a major cargo of this complex, and cells lacking CD47 have elevated levels of specific m^7^G-capped RNAs in their cytoplasm, but also globally higher levels of m^7^G-capped RNAs released in EVs. The lower basal levels of these m^7^G-capped miRNAs in WT cells and EVs are increased following treatment with TSP1. Thus, TSP1 can be viewed as a ligand that reverses a basal inhibitory function of CD47. Beyond increasing the nuclear localization of exportin-1, however, many details of the mechanism by which CD47 regulates the exportin-1/RanGTP complex remain to be determined. The GTPase activator RanBP1 and the regulatory binding protein RanBP3 also regulate nuclear-cytoplasmic shuttling of exportin-1 complex (Plafker and Macara 2000). Co-immunoprecipitation indicates that RanBP1, RanBP3, and RanGAP1 interactions with CD47 are not significantly GTP-dependent. However, this conclusion may apply only for the specific cell-free conditions studied. We found that the CD47 interaction with ubiquilin-1 in cytosolic extracts was decreased in response to GTPγS and LMB treatments. However, effects of GTPγS in cell extracts may not replicate regulation of Ran GTPase function in an intact cell. Further investigation will be required to determine which proteins interact directly to mediate the observed signaling responses and how GTP binding to Ran modulates these protein–protein interactions beyond the previous reported GTP induction of ubiquilin-1 association with exportin-1 (Kirli et al. 2015).

Another open question concerns where in the cell CD47 interacts with the exportin-1/RanGTP complex. We demonstrate that CD47 is present on a subset of vesicles in MVBs, but it is unclear whether CD47 regulates the RNA content of all EVs or only those expressing CD47. Inhibiting exportin-1 function using LMB moderately decreased the level of CD47 at the plasma membrane. This could reflect increased internalization of CD47, possibly leading to its degradation. However, LMB also increased the CD47 and exportin-1 released from cells in EVs. Does this CD47 control the uptake of m^7^G-capped RNAs that are subsequently released by the cells? Does CD47 also regulate the function of the exportin-1/RanGTP complex after EVs are released from cells?

EVs are known to be enriched in a cell-specific manner with miRNAs and other non-coding RNAs with compositions that differ from their parental cells (Que et al. 2013; Villarroya-Beltri et al. 2013; Zoller 2013; Cheng et al. 2014; Chevillet et al. 2014). Several RNA-binding proteins have been identified that regulate sequence-specific miRNA sorting (Mittelbrunn et al. 2011; Villarroya-Beltri et al. 2013; Zietzer et al. 2020), but mechanisms by which specific miRNAs are sorted into different subsets of EVs remain unclear. In retrospect, a report that exportin-1 function is required for the redistribution of high mobility group box-1 from the nucleus to cytoplasm and its release in EVs by macrophages (Chen et al. 2016) supports the broader function we identify here for the exportin-1 complex in regulating the m^7^G-capped RNA content of EVs. Our results are also consistent with an earlier report that the exportin-1 complex mediates the active packaging of the major HIV1 trans-activation response element (TAR) miRNA into exosomes released by infected cells (Narayanan et al. 2013).

The relevance of m^7^G-cap-dependent RNA trafficking to the physiological functions of CD47 in cardiovascular disease, aging, cancer, and infection remain to be investigated. Because the transcriptional response associated with T cell activation involves many m^7^G-capped RNAs (Schutz et al. 2006), regulation of capped RNAs by TSP1 may contribute to its CD47-dependent inhibition of T cell activation (Kaur et al. 2011; Miller et al. 2012, 2013). Furthermore, the CD47-dependent enrichment of such RNAs in EVs could contribute to differential effects of EVs from CD47^+^ and CD47^−^ cells on the activation of recipient T cells (Kaur et al. 2014b). The known roles of the exportin-1 pathway in viral pathogenesis may also be relevant to our recent report that clearance of a chronic LCMV infection is delayed in *Cd47*^*−/−*^ mice (Nath et al. 2018). NFAT nuclear export is mediated by this pathway (Kehlenbach et al. 1998), and impaired NFAT translocation in CD8^+^ T cells was identified as a cause of impaired clearance of a chronic LCMV infection (Agnellini et al. 2007).

## Supplementary Information

Below is the link to the electronic supplementary material.

Supplementary file1 (PDF 5589 KB)

## Data Availability

miRNA sequencing and microarray analysis-related raw data files are in supplementary datasets S1-S4 and deposited at GSE132646 and GSE168187. GSEA analysis of miRNA and mRNA is in datasets S5-S6. Mass spectrometry data for CD47 interacting proteins are available Dataset S7 and deposited at ftp://MSV000086969@massive.ucsd.edu. All data is contained in the manuscript or the indicated public databases

## Abbreviations

EIF4E: eukaryotic translation initiation factor 4E
EVs: extracellular vesicles
GTPγS: guanosine 5′-[γ-thio]-triphosphate
LMB: leptomycin-B
m^7^G: 5’-7-methylguanosine-modified
MVB: multivesicular bodies
RanBP1: Ran binding protein-1
RanBP3: Ran binding protein-3
RanGAP1: Ran GTPase activating protein-1
RIP: RNA immunoprecipitation
TSP1: thrombospondin-1
WT: wild type
XPO1: Exportin-1
