# Supplementary material for: CD47 interactions with exportin-1 limit the targeting of m7G-modified RNAs to extracellular vesicles
Source: J Cell Commun Signal. 2021 Nov 29;16(3):397–419. doi: 10.1007/s12079-021-00646-y (PMC9411329; doi:10.1007/s12079-021-00646-y)
Supplement: Supplementary file 1 — Supplementary file1 (PDF 5589 KB) [file 12079_2021_646_MOESM1_ESM.pdf]

Kaur et al, CD47 interactions with exportin-1 regulate targeting of m^7G-modified RNAs to extracellular vesicles

Extended Material and Methods

Isolation of EVs via Precipitation:

WT and $CD47^-$ Jurkat T cells ($25x10^6$) were cultured using HITES medium (90% Ham's F-12, 10% RPMI 1640 medium containing 5 mM HEPES, 2 mM glutamine, 0.1% BSA, 5 µg/ml insulin, 5 ng/ml sodium selenite, 5 µg/ml transferrin (Thermo Fisher Scientific, Waltham, MA), and 200 nM hydrocortisone (Sigma Aldrich, St. Louis, MO) for 24-48 h at 37°C. The cells were harvested by centrifugation at 1300 RPM for 5 minutes. Cell pellets were washed with 1X PBS for 5 minutes and used for total RNA extraction. Cell supernatants were collected into new tubes and centrifuged at 395xg (1300 RPM) for 5 minutes. Cell supernatants were collected into new tubes and centrifuged for 10 minutes at 2100xg using a Thermo Sorvall Legend XTR centrifuge (3000 RPM). The supernatants were concentrated using Centricon-YM-10 filters to 500 µl. The EVs were precipitated using the Exo-quick kit (SBI System Bioscience, Palo Alto, CA) as described (Kaur et al., 2014b;Kaur et al., 2018).

Size exclusion chromatography (SEC) method of EVs purification: Supernatants from WT and $CD47^-$ Jurkat T cells were prepared as described as above, and Exo-spin™ kit (Cell Guidance Systems, St. Louis, MO) used according to the manufacturer's instructions.. In brief, the concentrated supernatants were further centrifuged using Thermo Sorvall Legend XTR centrifuge (14000 RPM=~16,000 × g) for 30 minutes. Centrifuged supernatants were added to Exo-spin™ Buffer in a 2:1 ratio and kept at 4°C overnight. Precipitated EVs were collected and purified using Exo-spin™ column for direct total RNA and/or miRNA for real time PCR analysis. For immunocapture analysis, total precipitated EVs were used to capture all sizes of EVs.

Purification of EVs by Differential Ultracentrifugation: EVs were purified using ultracentrifugation Basic Protocol 1 according to (Thery et al., 2006) via sequential centrifugation for 10 min at 300xg to pellet cells, 10 min 2000xg to pellet debris, 30 min 10,000xg to pellet apoptotic bodies,1h 10 min at 100,000xg to pellet EV, and washed again at 100,000xg for 70 min. Total RNA was extracted using the TRIzol method (Thermo Fisher Scientific).

Purification of EVs via Density Gradient Ultracentrifugation: EVs purified via Ultracentrifugation can form aggregates with non-EV complex at high speed. Therefore, three-stage purification procedure as described in (Shurtleff et al., 2016) used. In brief, EVs were isolated using Exo-spin™ - Cell Guidance Systems and added to a volume up to 1 ml to the sequentially overlaid onto 60% (1 ml), 40% (1 ml) and 20% (1 ml) of Optiprep gradient solutions (Sigma, St. Louis, MO) according (Shurtleff et al., 2016). The tubes were centrifuged for 16 h in a T-880 rotor at 4^0C for 150,000xg (39,900 rpm). After centrifugation the fraction between 20% and 40% Optiprep layers was recovered into Eppendorf tubes.

Isolation of EVs via immune-capture: WT and CD47- T cells were cultured in HITES media, centrifuged and supernatants were collected as described earlier. The EVs were precipitated either using Exo-spin Kit Buffer A or purified via Density Gradient Ultracentrifugation. Equal amount of EVs with respect to their

protein contents (Pierce™ BCA Protein Assay Kit - Thermo Fisher Scientific) were diluted with 1XPBS and subjected to immune-capture using exportin-1, RANBP1, RANBP3, RANGAP1, ubiquilin-1, CD47 antibodies with immunoconjugated super magnetic beads overnight at 4°C. The immunocapture EVs were washed three times with 1XPBS and used for downstream analysis such as western blot, RNA-immunoprecipitation, and RT-PCR etc.

NanoSight Analysis: A NanoSight NS300 was used for nanoparticle characterization according to manufacturer's instructions as described previously (Kaur et al., 2018).

Nanoview Analysis: EVs from WT and CD47- T cells were cultured in HITES media and purified using Exo-spin™ columns as described above. EV samples were diluted (1:10), and 35 µL volumes were incubated on chips overnight. The next day, chips were washed to remove unbound material, and chips were incubated with primary antibodies for 1 hour and washed 4 times. The chips were rinsed, dried, and imaged in an ExoView R100 (Nanoview Biosciences).

Negative Stain Electron Microscopy:

Purified EV samples were adhered to freshly glow-discharged, formvar and carbon coated, 300 mesh copper EM grids (Electron Microscopy Sciences, EMS) by inverting the grid on a 5 µl drop of sample placed on Parafilm for one minute. Grids with adhered sample were transferred across two drops of 2% aqueous uranyl acetate (UA) for one minute. The grid was then picked up and carefully blotted with filter paper, allowing a thin layer of UA to dry on the grid.

Samples to be immunogold labeled were adhered to EM grids, passed through 20 µl drops of filtered PBS to rinse away unbound sample, and then incubated on drops of blocking solution containing 3% BSA (Sigma) in PBS for 30 minutes, to reduce non-specific antibody binding. Samples were covered during incubation steps to prevent evaporation. Primary antibody to CD47 (clone CC2C6, BioLegend) was diluted in filtered blocking solution to a working concentration of 20 µg/mL. After blocking, grids were blotted lightly with filter paper to remove excess solution before transfer to primary antibody droplet, and then incubated for 60 minutes. Then, grids were transferred across two drops of blocking solution to rinse away unbound primary antibody and incubated for 20 minutes to block before incubation with secondary antibody. Secondary antibody (10 nm colloidal gold-conjugated goat anti-mouse IgG; EMS) was diluted 1:25 in filtered blocking solution. Grids were lightly blotted before transfer to droplet of secondary antibody and incubated for 30 minutes. Then, grids were rinsed in 3 drops of PBS. Prior to negative stain, grids were quickly transferred to a drop of distilled water to remove PBS before negative staining as described above.

Samples were observed using a Technai T20 transmission electron microscope operated at 200 kV. Images were collected using an AMT NanoSprint1200 CMOS detector (Advanced Microscopy Techniques, Woburn MA).

Quantification of miR expression and copy number

miR expression and Copy number was calculated according to (Zietzer et al., 2020). In brief, 20 µl reverse-transcription reaction was prepared using 5 µl miScript miRNA Mimic (10^{10} copies/µl) and 50 ng bacterial RNA extracted from E.Coli (DH5α Competent Cells from Thermo Scientific) was used as carrier. Reaction was Incubated for 60 min at 37ºC and miScript Reverse Transcriptase was inactivated by incubating at 95ºC for 5 min and on ice at 4°C.480 µl carrier bacterial RNA (1 ng/µl concentration) was added to the 20 µl reaction. The reaction tube was mixed gently by pipetting up and down and centrifuged briefly. This dilution yields 10^8 copies cDNA/µl (assuming an efficiency of 100%). Using the diluted cDNA mix from this step and carrier bacterial RNA (1 ng/µl), prepare serial dilutions. Template copy numbers in the PCRs range from 6x 10^{10} to 6x 10^5 was generated. PCRs used to plot a standard curve of CT values (y axis) against log copy number (x axis). The Ct value (X) of WT and $CD47^-$ cells and their EVs were calculated using (slope*X+yint) corresponding to the standard curve of mimics-320a.

Visualization and re-analysis of T cell EVs on target HUVEC gene expression: The data sets for mRNA differentially expressed between EVs from WT and $CD47^-$ (Dataset S2) and RNAs differentially expressed in WT and CD47-deficient EVs (Dataset S3) were extracted from previously published (Kaur, 2014b, Matrix Biology) for re-analysis and visualizations for Venn Diagrams and miRNA target prediction analysis.

mRNA target prediction in endothelial cells for CD47-dependent miRNAs in EVs: Differentially expressed miRNAs (~10 miRNAs up down) from each data sets (Data S1 and Data S2A EVs-WT _ CD47-control), and ~10 miRNAs which are common between these two sets were used for miRNA target prediction analysis. miRNA targets were identified using either miRDB or TargetScan- MicroRNA Target Prediction Database, and then compared with WT and $CD47^-$ EVs microarray analysis from (Data S2B EV miRNA HUVEC targets) using FunRich V3 software. The predicted mRNA -miRNA targets were aligned with mRNA induced in recipient endothelial cells induced by treatment EVs derived from WT versus CD47-T cells (Data S2B EV miRNA HUVEC targets).

CD47 EVs in the presence of LMB: Jurkat T cells were treated with 20 nM of LMB (cell signaling) for 2 hrs. Cell supernatants were collected into new tubes and centrifuged for 10 minutes at 395xg for 5 minutes. Cell supernatants were collected into new tubes and centrifuged for 10 minutes at 2103xg using a Thermo Sorvall Legend XTR. The supernatants were concentrated and precipitated using Exo-Spin kit buffer overnight according to manufactures' instructions. The precipitated EVs were solubilized, using LDS in PBS Buffer containing Triton X-100 and were boiled at 95°C for 5 minutes. The EVs were subjected to western blot analysis using 4-12% Bis-tris gel. CD47 antibody (CC2C6) was used to detect CD47.

Total EVs extraction for western blots: WT and $CD47^-$ T cells were treated with 20 nM of LMB (Sigma or Cell Signaling) for 2 h. Cell pellets were washed with 1X PBS for 5 minutes and used for total RNA extraction. Cell supernatants were collected into new tubes and centrifuged for 10 minutes at 395xg for 5 minutes. Cell supernatants were collected into new tubes and centrifuged for 10 minutes at 2103xg using a Thermo Sorvall Legend XTR. The supernatants were concentrated and precipitated using Exo-spin™ buffer (Cell Guidance Systems) overnight according to manufactures' instructions. The precipitated EVs were solubilized, and exportin-1-immunoprecipitation (Proteintech, Rosemont, IL) and western blots were

performed using CD47 (Santa Cruz Biotechnology, Inc. Dallas, Texas) RANBP1, RANBP3, RANGAP1, ubiquilin-1 (Cell Signaling, Danvers, MA) and exportin-1 antibodies (Proteintech).

EV-Subset isolation with TSP1 treatment: WT and CD47$^-$ T cells were treated with 20 nmol of LMB (Sigma or Cell Signaling) and TSP1 (1 µg/ml) as indicated using HITES medium for 1-2 h at 37°C. The cells were harvested using centrifuge at 1300 RPM for 5 minutes. Cell pellets were washed with 1XPBS for 5 minutes and used for total RNA extraction or protein fractions. Cell supernatants were collected into new tubes and centrifuged for 10 minutes at 395xg (1300 RPM) for 5 minutes. Cell supernatants were collected into new tubes and centrifuged for 10 minutes at 2103xg using Thermo Sorvall Legend XTR. The cell superannuates were concentrated and centrifuged for 10,000xg for 10 minutes using a 5417R centrifuge (Eppendorf). The pellets were collected and used for western blots only. The supernatants were precipitated using Exo-Spin kit buffer overnight according to manufacturer's instructions. The precipitated pellet was resuspended into PBS, and exportin-1-immunoprecipittaion and western blotting were performed using RANBP1 and exportin-1 antibodies.

Phenotype analysis of LMB treatment via confocal analysis: WT and CD47$^-$ T cells were incubated in HITES medium overnight. Next day, the WT and CD47- T cells were plated on poly-lysine coated 4-well bidi chambers (bidi USA, Inc.). and treated with LMB (20 nM) for 1 hour. The cells were fixed with 4%PFA (Sigma Aldrich, USA) and immunostained with exportin-1 (Santa Cruz Biotechnologies, USA) cells as described earlier (Kaur et al., 2014a). The cells were fixed using VECTASHIELD® Hardset™ Antifade Mounting Medium with Phalloidin (VECTOR LABORATORIES, INC). The confocal images were captured using Zeiss LSM 780 microscope with a 63x objective (Plan-Apochromat, 1.40NA). Cell boundaries in white were drawn based upon phase contrast imaging. exportin-1 signal inside the cell boundary was pseudo-colored in red, while signal outside the cell boundary was pseudo-colored in green. Scale bar = 5 µm.

Western Blotting: Jurkat and JinB8 cells were treated with LMB (20 nM) for 1 hour. Cell cultures were harvested and centrifuged to obtain conditioned media. The cells were washed with cold PBS and lysed with RIPA Buffer (Thermo-Fisher) for total lysates, or with NE-PER (Thermo-Fisher) for cytoplasmic and nuclear lysates. A BCA protein assay (Pierce) was performed in order to quantify total protein. For each sample, we loaded 20 µg of protein with the appropriate amount of LDS buffer (Invitrogen) into the gels. Gels were electrophoresed at 200 V and transferred onto PVDF membranes using the Invitrogen iBlot system and transfer stacks. Membranes were blocked with 3-5% BSA for one hour, incubated with primary antibodies overnight at 4°C, incubated with secondary antibody for 1 hour at room temperature, and imaged on a Bio-Rad Chemidoc MP system

Thin section electron microscopy with immunogold labeling for CD47: Pre-embedding immunogold labeling of Jurkat T cells (Clone E6-1 (ATCC® TIB-152™): Reagents were obtained from Electron Microscopy Sciences and steps conducted at room temperature unless otherwise stated. Jurkat T cells were pelleted at 1000 g for 5 minutes and resuspended in medium to a concentration of 1 x 10^6 cells/mL. Cells in suspension were treated with 20 nM LMB (Cell signaling) or vehicle control (ethanol) for 30 minutes

in the incubator before being adhered Aclar coverslips that were previously coated with monoclonal antibody to integrin (clone TS2/16). To prepare coverslips, Aclar was cut to fit into wells of a 12-well plate, rinsed with distilled water, ethanol, and then dried. Droplets of TS2/16 antibody (5 ug/mL in PBS) were placed onto each coverslip and incubated at 4° C for 6 hours. To adhere cells, the antibody was withdrawn, the area rinsed once with sterile PBS (Gibco) and replaced with a droplet of cell suspension for 30 minutes in the incubator. Total duration of LMB or vehicle treatment time was 60 minutes. Adhered cells were then fixed with 4% EM-grade paraformaldehyde in 0.15M Sorensen's phosphate buffer (PB) for 60 minutes. After fixation, cells were rinsed thrice with PB and twice with PBS, followed by 15 minutes incubation in PBS containing 50 mM ammonium chloride (Sigma). After two more PBS rinses, cells were blocked and permeabilized with 0.1% saponin (Sigma) and 2% BSA (Sigma) in PBS (blocking solution) for one hour. Cells were incubated in biotinylated mouse anti-human CD47 (BioLegend, clone CC2C6) diluted in blocking solution to 2 μg/mL for 60 minutes. Cells were rinsed twice in blocking solution for 20 minutes, and then cells were incubated with nanogold conjugated goat anti biotin antibody (Nanoprobes, #2015) diluted 1:100 in blocking solution for 60 minutes. Cells were rinsed twice with blocking solution, twice with PBS and twice with PB before post fixation in 2% EM grade glutaraldehyde in PB for 15 minutes. After fixation, cells were rinsed with PB and quenched with 50 mM ammonium chloride in PB for 15 minutes. To silver intensify nanogold, cells were rinsed thoroughly with distilled water and then silver intensified using HQ Silver enhancement kit (Nanoprobes, #2012) according to manufacturer's instructions. After extensive rinsing with distilled water, samples were gold toned to protect silver particles from etching by subsequent heavy metal staining (Arai et al., 1992) . Cells were placed on ice and incubated in 0.05% gold chloride for 10 minutes at 4° C. After several rinses with water, cells were incubated in 0.5% oxalic acid for 2 minutes and then rinsed thoroughly with water and then twice with PB. Cells were stained with potassium ferrocyanide-reduced osmium (0.25% osmium and 6 mM potassium ferrocyanide in PB) for 30 minutes, then rinsed with PB before incubation in 0.5% tannic acid in PB for 30 minutes. Cells were then rinsed with thrice with PB and then rinsed thoroughly with acetate buffer (pH 5.2) before 30 minutes incubation in 0.5% uranyl acetate in acetate buffer. Cells were rinsed in acetate buffer three times, and then dehydrated with a graded series of ethanol rinses and embedded in EMBed-812 embedding medium by inverting resin filled capsules over the cells. For ultrathin sectioning, Aclar coverslips were removed and 70-nm thick sections were cut in an en face orientation. Sections were collected on slot grids and contrasted with 1% uranyl acetate followed by Reynolds lead citrate. Sections were examined using a ThermoFisher-FEI Technai T20 transmission electron microscope operated at 80 kV and images collected with an AMT Nanosprint12 CMOS camera. Images for display were smoothed with a 0.5-pixel gaussian blur and adjusted for brightness and contrast using Adobe Photoshop 2020.

Flow Cytometry Analysis: CD47 expression was measured using CD47 antibodies (Biolegend) and BD-intracellular kit (BD Biosciences, MD, USA) according to manufactures instructions as described earlier (Kaur et al., 2014a). WT cells were treated with LMB for 1 h and cell surface CD47 was analyzed using primary anti-human CD47 (Proteintech) for 30 min at room temperature. Cells were washed with FACS

buffer (HBSS containing 4% FBS) and incubated with FITC-conjugated anti-mouse antibody (eBiosciences) for 30 min at room temperature. In addition, cell were also stained with anti-human CD47 with FITC-secondary antibody or CD47-APC (Biolegend). Samples were acquired on an LSR Fortessa cytometer (BD Biosciences) and analyzed using FlowJo software.

Supplemental Figure Legends

Fig S1. WT and CD47$^-$ EV characterization. (A-D) Electron micrographs of extracted EVs (Exo-spin™) from resting T cells, Scale bars = 200 nm (A, B) and activated WT and CD47$^-$ T cells Scale bars = 100 nm (C, D). Electron microscopic analysis of EVs derived from unstimulated WT and CD47$^-$ cells (A, B) as well as cells activated with anti-CD3 and anti-CD28 showed similar cup shaped morphologies (C, D). (E, F) Extracted EVs (Exo-spin™) size and concentration were measured using NanoSight analysis. (G, H) Extracted EV (Exo-spin™) size and surface protein expression of CD63, CD81, CD9 and CD41 (Tetraspanins family) were examined using ExoView® R100 instrument. Co-localization of CD47 and tetraspanins on EVs was examined using Exoview chips, (I-J) represent label free EVs count and (K-L) CD47 positive EVs bound to chips. (M) Validation of cell surface and intracellular CD47 protein expression via flow cytometry and confocal microscopy using WT and CD47$^-$ T cells.

Fig S2: CD47-dependent miRNA enrichment in EVs and validation of CD47$^-$ cells by CRISPR knockdown and CD47 re-expression. (A) Genes differentially expressed in EVs released from WT and CD47$^-$ T cells based on microarray analysis (p value<0.05 and >1.2-fold change) were analyzed by class (B) and hierarchical clustering . (C) Venn diagram comparing CD47-dependent mRNAs in T cell EVs and CD47-dependent mRNAs in human umbilical vein endothelial cells treated with EVs derived from WT versus CD47$^-$ Jurkat cells. Only one mRNA in the EVs matched those regulated in endothelial cels treated with the same EVs. (D) Validation of microarray and miRNA sequencing results using CRISPR/Cas9 mediated loss of CD47 function in WT Jurkat T cells. (E-F) EVs collected from CD47- T cells stably transfected with control (VEC) or CD47 expression plasmids (CD47+) were analyzed for expression of the indicated miRNA using real-time PCR. The expression was normalized to EVs extracted from WT Jurkat cells using B2M as control. Significant values were determined using the default t-test of CFX Realtime PCR software from Bio-Rad.

Fig S3. (A-D) Immunoprecipitation of CD47 and western blots for ubiquilin1, RanGAP1, RanBP1 and exportin-1 in lysates from untreated cells and treated with LMB or GTPγS. (A,B) Immunoprecipitation of CD47 and western blots for RanBP3 in the presence of LMB and GTPγS (C,D) quantification of relative intensity of western blots for RanBP1 and RanBP3, and statistical analysis was performed using two-way ANOVA**.** (E) Protein expression of exportin-1 on EVs derived from WT and CD47$^-$ EVs isolated using density gradient centrifugation. (F) LMB treatment induced exportin-1+ (green) vesicle release as compared to untreated cells. Scale = 5 μm. Number of cells analyzed: WT cells (UT=372, LMB=524), The statistical significance of the vesicle phenotype was calculated using Two-Sample Assuming Equal Variances t-test

(UT=22, LMB=102). * = p< 9.56302E-11. (G) Confocal microscopy of WT and CD47- T cells treated with 20 nM LMB for 1 h and immunostained using exportin-1 antibody. (H) Manual quantification of cytoplasmic expression of exportin-1 by comparing WT and CD47- T cells and t-test: assuming Two-Sample Assuming Equal Variance. Number of cells analyzed: WT cells (UT=23, LMB=72), CD47- cells (UT=10, LMB=140). * = p< 0.005. (I) cell surface staining of WT T cells treated with 20 nM LMB for 1 h using CD47 antibody from Proteintech with FITC secondary (upper panel) and direct staining with anti-CD47-APC (Biolegend, lower panel). (J) Confocal microscopy of WT T cells treated with 20 nM LMB for 1 h and immunostained using anti-CD47. (K) Western blot analysis of CD47using EVs derived from Untreated and LMB treated Jurkat T cells

Fig S4: A-L) Immunogold label for CD47 in Jurkat cells treated with vehicle (A, C- E) or LMB (B, F-K). (A) whole-cell views of the cells that are enlarged in Fig 5A and B, respectively. (C-E) Additional examples of vehicle (C-E) and LMB (F-H) treated cells. Boxed area in C and F is shown in D and G; arrowheads indicate label on cell surface. Labeled MVB in D is enlarged in E, labeled MVB in G is enlarged in H. (I-K) show labeling on a cell in which primary antibody to CD47 was omitted. Label is absent on the cell surface (J) and absent in MVBs (K). Sparse non-specific label occurred in the cytoplasm, predominantly associated with mitochondria. (L) A montage of 6 additional MBVs with label for CD47. Scale bars in A, B, C, F and I = 2 µm; scale bars in D, G and J = 1 µm; scale bars in E, H, K and L = 200 nm. (M) Immunoprecipitation of exportin-1 was performed using EVs derived from WT and CD47- treated cells with 20 nM LMB, and western blot analysis was performed using RanBP1, RanGAP1, RANBP3 and exportin-1 antibodies. (N,O) precursor miRNAs differentially expressed between WT cells vs EVs (Red) and $CD47^-$ cells vs EVs (Red) (Data S2B, p<0.05) were compared with data set RIP-m^7G-capped miRNAs (1-s2.0-S1097276519302692-mmc5) from (Pandolfini et al., 2019) which show common m^7G-capped miRNAs in WT EVs vs cells (273) and CD47 EVs $^-$ vs cells (340) (p<0.05). For both comparisons for WT EVs vs cells (Red) and $CD47^-$ EVs vs cells (Red) the parameters used to create data: Technology = Whole transcriptome RNA-Seq, Test differential expression due to = sample, Comparisons = Against control group, Control group = WT or $CD47^-$ cells.

Fig S5. (A) WT and $CD47^-$ T cells were transferred into serum free HITES medium for 24 h. EVs were isolated and separated using density gradients as indicated, and miRNA-m320a-3p (Quanta Bioscience), pre-miR-34a and pre-miR-125b were analyzed using real time PCR from total EV-RNAs. EVs released by WT and CD47- cells were isolated by density gradient centrifugation for 16 h, lysed, and expression of the indicated miRNAs analyzed. (C) Standard curve of mimic 320a using *E.coli* RNA as carrier via miScript miRNA PCR Assay for mature miR-320a. (D) Calculation of copy number of miR320a using Ct values from Fig 3 G&H. CD47- and activity-dependent interactions of exportin-1 with miRNAs was analyzed using (E) amount of INPUT (Untreated $CD47^-$ compared with untreated WT using U6. Ct. miR320a-3p (WT: 36.21± 0.33, $CD47^-$: 34.41±0.13), miR34a (WT: 39.55±0.28, $CD47^-$: 34.87±0.12), miR125b (WT: 37.05±1.5, $CD47^-$: 33.31±0.22),U6 (WT: 22.94±0.92, $CD47^-$:20.44±0.29) (F-H) Enrichment of exportin-1 dependent pre-

miRNA. using RNA-immunoprecipitation of exportin-1 using WT and $CD47^-$ T cells were compared to UT control depicted from Fig (3F-H).

Fig S6. (A) Confocal image analysis of Jurkat T cells treated with LMB (20 nM) and/or TSP1 (1 μg/ml) and then stained with anti-exportin-1 and anti-RANBP1. Scale bar= 5 μm. Effects of TSP1 and/or LMB treatment on components of the exportin-1 complex released into EVs from Jurkat T cells. (B) Flow chart depicting method of EVs extraction and lysate preparation. (C) Western blot analysis of EV subsets isolated by differential centrifugation as shown. (D) Quantification of RanBP1 (E) Quantification of exportin-1 relative to Coomassie blue staining.

Fig S7. List of Real-Time PCR primers (human and mouse) used in the manuscript were purchased from Integrated DNA Technologies, Inc.

List of Supplementary Data Sets cited in the text

1. **Data S1.** List of differentially expressed miRNAs (2-fold up/down and pval<0.05) in EVs derived from WT and $CD47^-$ T cells via Microarray Analysis using GeneChip™ miRNA 3.0 Array. Related to Fig 1A.
2. **Data S2A.** List Differentially expressed miRNAs, (mature and precursor miRNAs with pval<0.05) in WT (n=3) and $CD47^-$ T(n=2, 1 replicate was unable to map) cells via miRNA sequencing analysis . Parameters used to create data; Technology = Small RNA, Normalization method = TMM, Comparisons = Against control group, Control group = WT cells. List of differentially expressed miRNAs (mature and precursor miRNAs and pval<0.05) in EVs derived from WT (n=4) and $CD47^-$ T (n=3) cells via miRNA sequencing analysis. Parameters used to create data; Technology = Small RNA, Normalization method = TMM, Comparisons = Against control group, Control group = $CD47^-$ EVs. Related to Fig 1G, Fig S1E&F.
3. **Data S3C.** Differential expressed miRNAs validation criteria used for Fig 1H from data correspondence to Fig A,B,E and F .
4. **Data S2B.** Target analysis of miRNAs differentially expressed in EVs derived from WT versus $CD47^-$ Jurkat T cells. Column A: mRNAs differentially regulated in human umbilical vein endothelial cells by treatment with EVs derived from WT versus CD47- cells. Column B: miRNAs differentially expressed in EVs derived from WT versus CD47- cells that are predicted to regulate the mRNA in column A based on Target Scan or mirDB. Pubmed IDs reference documented roles for each miRNA in angiogenesis (column I) or functional effects on endothelial cells when delivered via EVs (Column K). Related to Fig 1.
5. **Data S2C.** seed/precursor miRNAs differentially expressed between WT cells vs EVs and $CD47^-$ cells vs EVs (p<0.05). The parameters used to create data; Technology = Whole transcriptome RNA-Seq, Test differential expression due to = sample, Comparisons = Against control group, Control group = WT cells or CD47- T cells. Related to Fig S1G , S1H and Fig1H.

6. **Data S3.** 113 Differentially expressed miRNAs (seed/precursors and mature), in WT and CD47- T cells and their EVs. 64 Differentially expressed miRNAs (seed/precursors and mature), in WT and CD47- T EVs only. Data of miRNA related to Fig1G. Among these EVs, overlapping percentage between XPO1 miRNA were calculated using data sets from (Castanotto et al., 2009;Martinez et al., 2017;Sexton et al., 2019). Percentage of common miRNA between M^7G-RIP and EVs were determined using data set 1-s2.0-S1097276519302692-mmc5 from (Pandolfini et al., 2019). Related with Fig 1G
7. **Data S4:** Ct values of miRNAs with B2M as control using (Vector) or CD47 re- expressed EVs in $CD47^-$ cells (A). Enrichment of m^7G-cap-dependent miRNAs in cd47-/- mouse plasma (B). Related with Fig 1E.
8. **Data S5.** GSEA Results Summary: using PMID28584122_EXPORTIN-1_ and CD47-WT_versus_CD47-KO dataset regulated_microRNAs in EVs. Related with Fig 2C.
9. **Data S6.** WT versus CD47- cells using a dataset of cap-dependent mRNAs identified in diffuse B cell lymphoma cells by RNA sequencing of nuclear EIF4E immunoprecipitates data from Bijana Blood 2016 . Related with Fig 3A.
10. **Data S7.** WT and $CD47^-$ cells were immunoprecipitated with streptavidin beads using biotin-conjugated CD47 antibody (CC2C6). CD47 Specific Mass spectrometry data was extracted by removing background data identified using $CD47^-$ cells. Related with Fig 4A.

REFERENCES

Arai, R., Geffard, M., and Calas, A. (1992). Intensification of labelings of the immunogold silver staining method by gold toning. *Brain Res Bull* 28**,** 343-345.

Castanotto, D., Lingeman, R., Riggs, A.D., and Rossi, J.J. (2009). CRM1 mediates nuclear-cytoplasmic shuttling of mature microRNAs. *Proc Natl Acad Sci U S A* 106**,** 21655-21659.

Enderle, D., Spiel, A., Coticchia, C.M., Berghoff, E., Mueller, R., Schlumpberger, M., Sprenger-Haussels, M., Shaffer, J.M., Lader, E., Skog, J., and Noerholm, M. (2015). Characterization of RNA from Exosomes and Other Extracellular Vesicles Isolated by a Novel Spin Column-Based Method. *PLoS One* 10**,** e0136133.

Joerger-Messerli, M.S., Oppliger, B., Spinelli, M., Thomi, G., Di Salvo, I., Schneider, P., and Schoeberlein, A. (2018). Extracellular Vesicles Derived from Wharton's Jelly Mesenchymal Stem Cells Prevent and Resolve Programmed Cell Death Mediated by Perinatal Hypoxia-Ischemia in Neuronal Cells. *Cell Transplant* 27**,** 168-180.

Kaur, S., Chang, T., Singh, S.P., Lim, L., Mannan, P., Garfield, S.H., Pendrak, M.L., Soto-Pantoja, D.R., Rosenberg, A.Z., Jin, S., and Roberts, D.D. (2014a). CD47 signaling regulates the immunosuppressive activity of VEGF in T cells. *J Immunol* 193**,** 3914-3924.

Kaur, S., Elkahloun, A.G., Arakelyan, A., Young, L., Myers, T.G., Otaizo-Carrasquero, F., Wu, W., Margolis, L., and Roberts, D.D. (2018). CD63, MHC class 1, and CD47 identify subsets of extracellular vesicles containing distinct populations of noncoding RNAs. *Sci Rep* 8**,** 2577.

Kaur, S., Singh, S.P., Elkahloun, A.G., Wu, W., Abu-Asab, M.S., and Roberts, D.D. (2014b). CD47-dependent immunomodulatory and angiogenic activities of extracellular vesicles produced by T cells. *Matrix Biol* 37**,** 49-59.

Martinez, I., Hayes, K.E., Barr, J.A., Harold, A.D., Xie, M., Bukhari, S.I.A., Vasudevan, S., Steitz, J.A., and Dimaio, D. (2017). An Exportin-1-dependent microRNA biogenesis pathway during human cell quiescence. *Proc Natl Acad Sci U S A* 114**,** E4961-E4970.

Pandolfini, L., Barbieri, I., Bannister, A.J., Hendrick, A., Andrews, B., Webster, N., Murat, P., Mach, P., Brandi, R., Robson, S.C., Migliori, V., Alendar, A., D'onofrio, M., Balasubramanian, S., and Kouzarides, T. (2019). METTL1 Promotes let-7 MicroRNA Processing via m7G Methylation. *Mol Cell* 74**,** 1278-1290 e1279.

Schindelin, J., Arganda-Carreras, I., Frise, E., Kaynig, V., Longair, M., Pietzsch, T., Preibisch, S., Rueden, C., Saalfeld, S., Schmid, B., Tinevez, J.Y., White, D.J., Hartenstein, V., Eliceiri, K., Tomancak, P., and Cardona, A. (2012). Fiji: an open-source platform for biological-image analysis. *Nat Methods* 9**,** 676-682.

Sexton, R., Mahdi, Z., Chaudhury, R., Beydoun, R., Aboukameel, A., Khan, H.Y., Baloglu, E., Senapedis, W., Landesman, Y., Tesfaye, A., Kim, S., Philip, P.A., and Azmi, A.S. (2019). Targeting Nuclear Exporter Protein XPO1/CRM1 in Gastric Cancer. *Int J Mol Sci* 20.

Shurtleff, M.J., Temoche-Diaz, M.M., Karfilis, K.V., Ri, S., and Schekman, R. (2016). Y-box protein 1 is required to sort microRNAs into exosomes in cells and in a cell-free reaction. *Elife* 5.

Thery, C., Amigorena, S., Raposo, G., and Clayton, A. (2006). Isolation and characterization of exosomes from cell culture supernatants and biological fluids. *Curr Protoc Cell Biol* Chapter 3**,** Unit 3 22.

Zietzer, A., Hosen, M.R., Wang, H., Goody, P.R., Sylvester, M., Latz, E., Nickenig, G., Werner, N., and Jansen, F. (2020). The RNA-binding protein hnRNPU regulates the sorting of microRNA-30c-5p into large extracellular vesicles. *J Extracell Vesicles* 9**,** 1786967.

Supplemental Figure S1

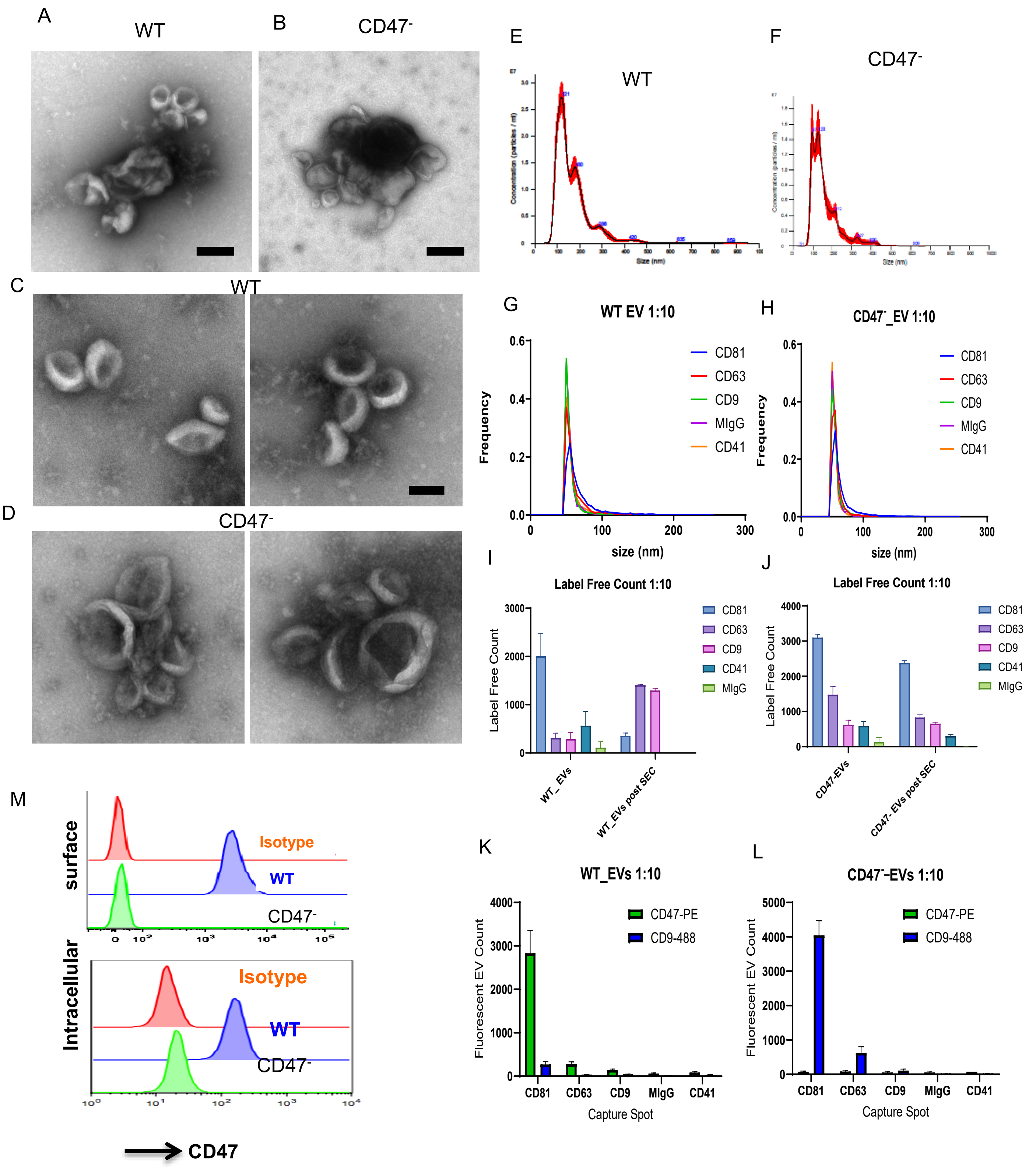

Supplemental Figure S2

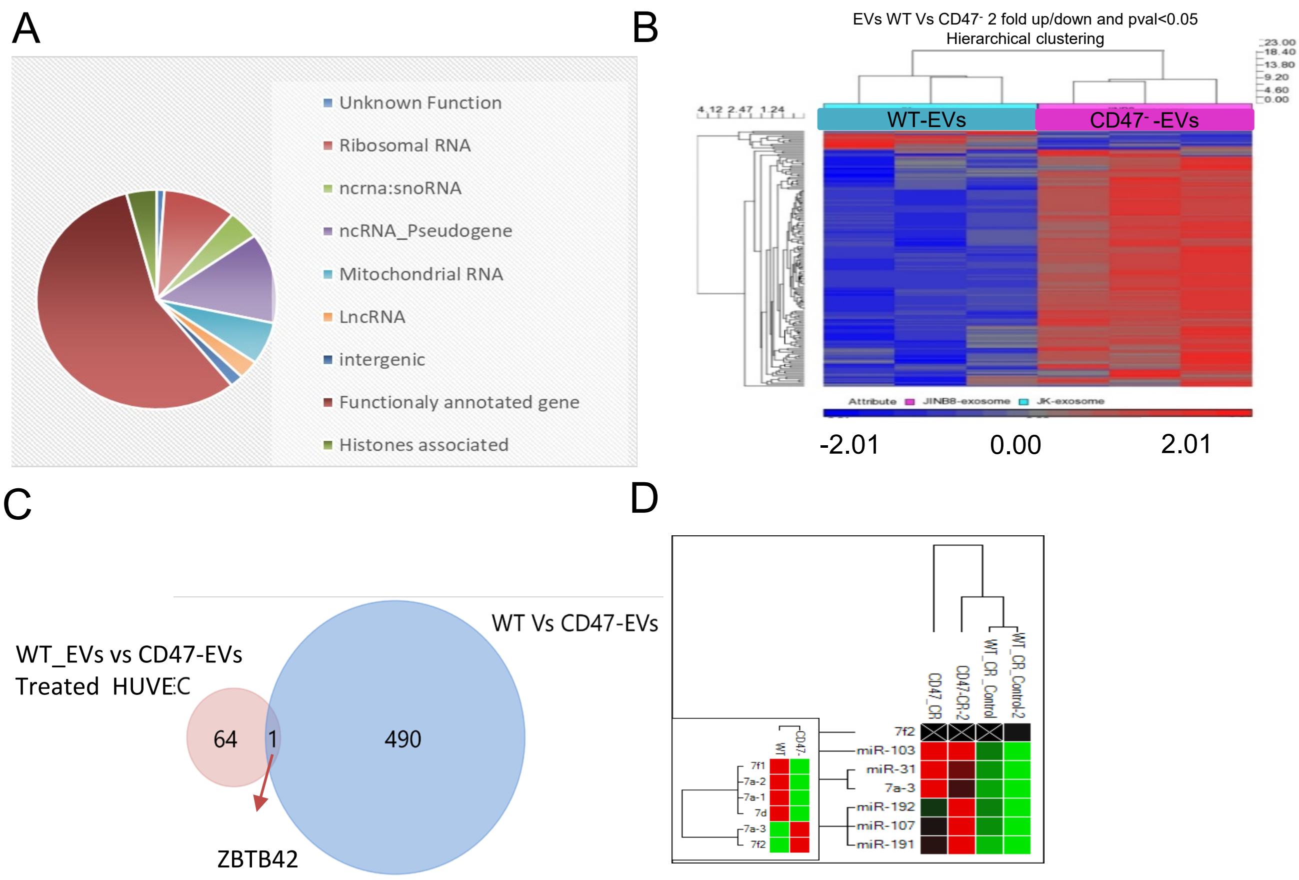

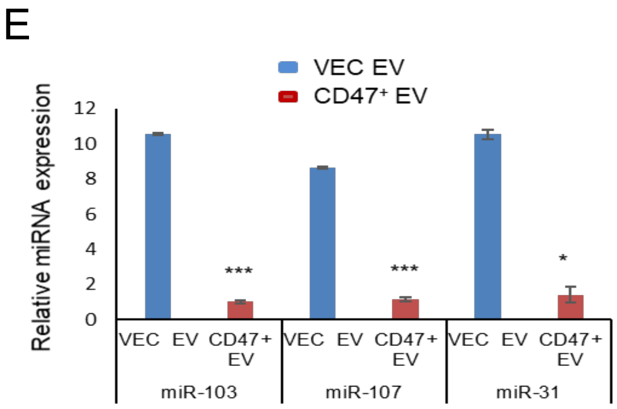

F

	Control (B2M)	Pre-MiR-125b	Pre-MiR-15b	Pre-MiR-34a	Pre-MiR-138	Pre-MiR-191
VECTOR-EV	20.44	26.56	25.13	28.8	28.63	31.19
	21.41	26.44	24.8	28.89	28.12	30.23
	20.79	26.71	25.37	29.01	29.16	31.11
CD47+-EV	19.69	30.61	25.1	34.13	34.94	32.92
	19.25	30.94	25.18	34.25	33.29	32.1
	19.37	30.56	25.78	33.14	34.63	33.05
Anova		≤ 0.0001	≤ 0.05	≤ 0.0001	≤ 0.0002	ns
t-test		≤ 0.0001	ns	≤ 0.0001	≤ 0.0006	≤ 0.01

Supplemental Figure S3

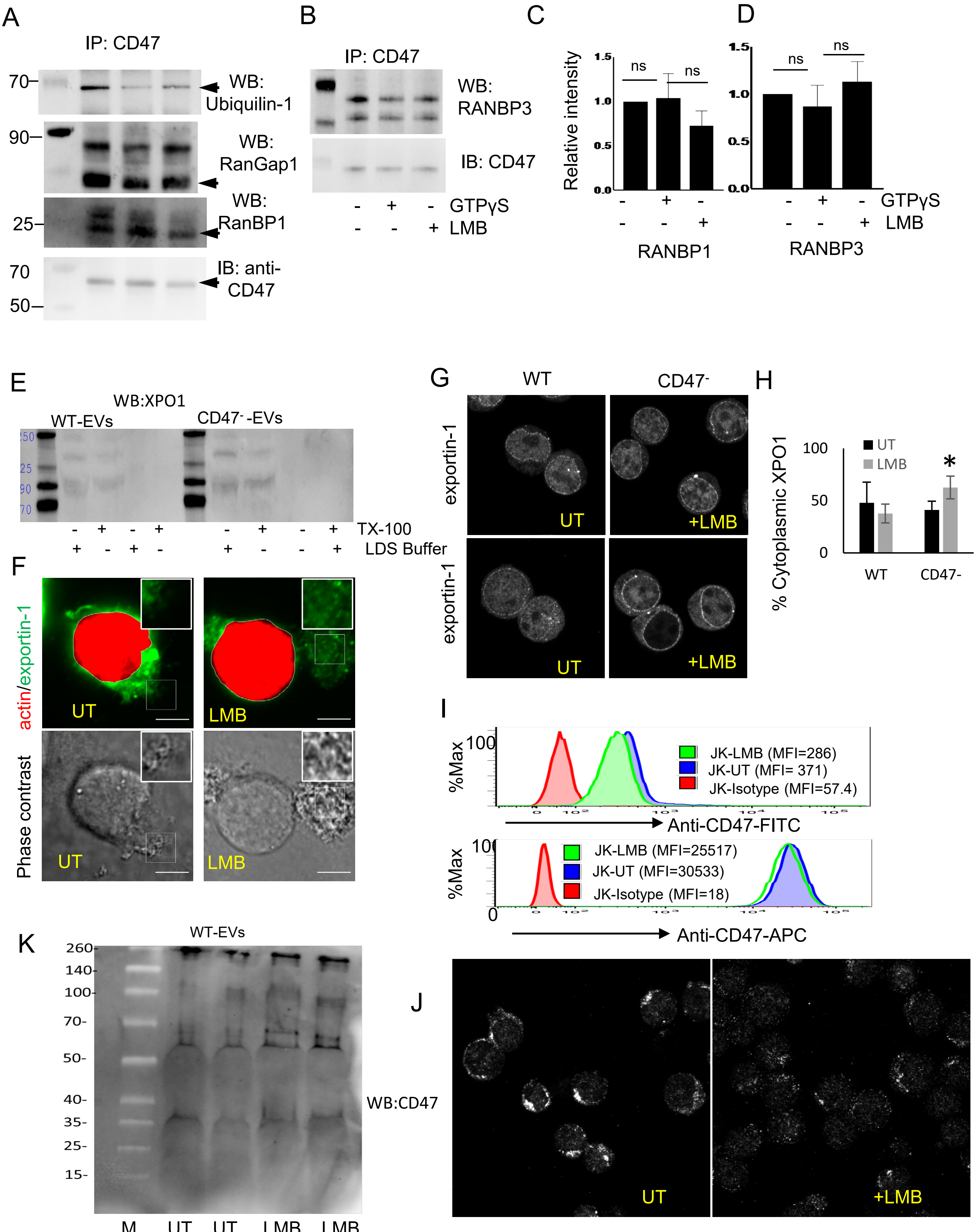

Supplemental Figure S4

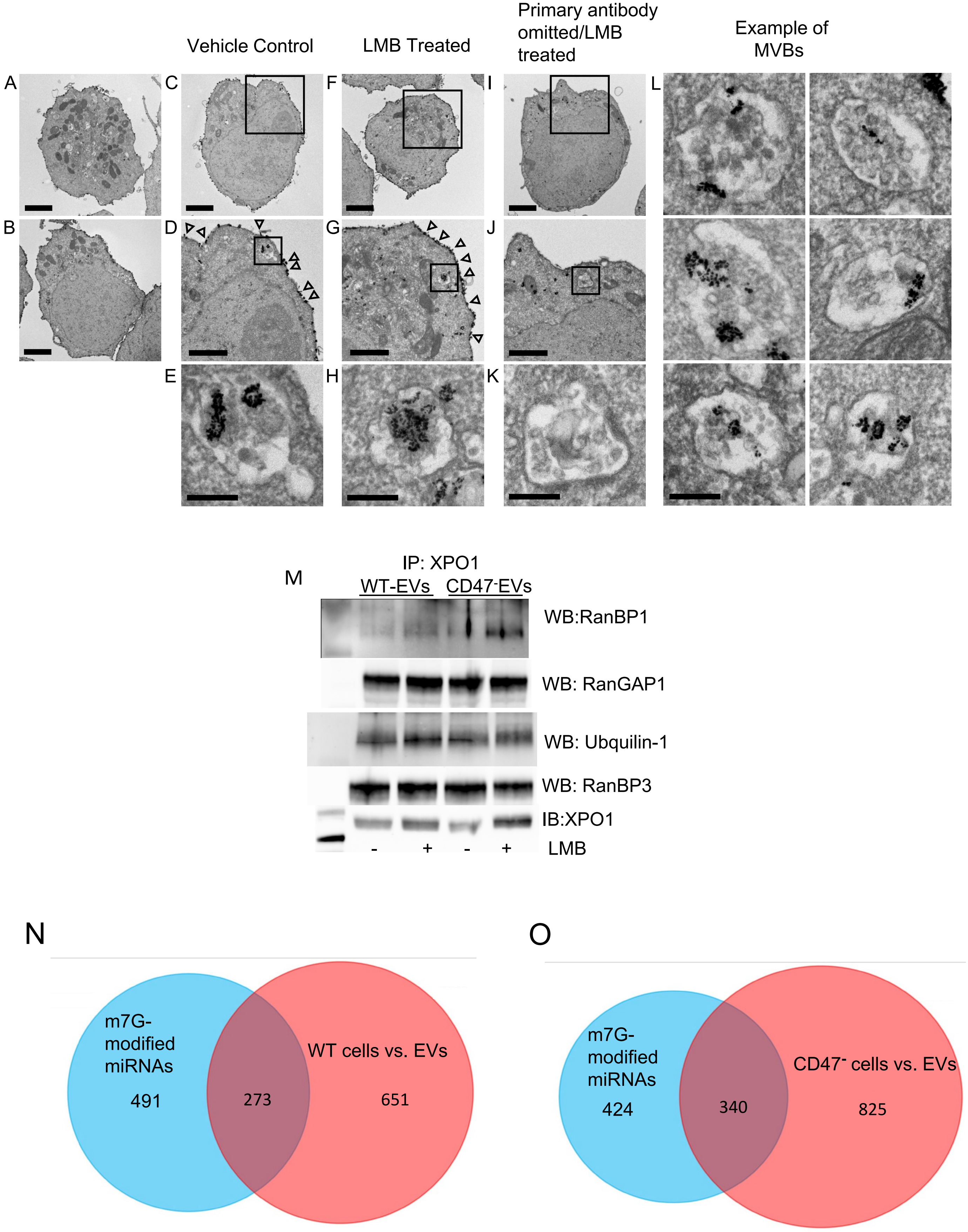

Supplemental Figure S5

A

A

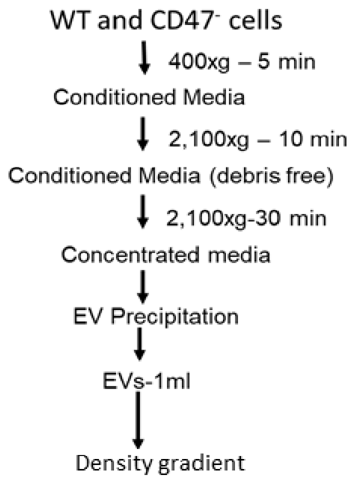

B

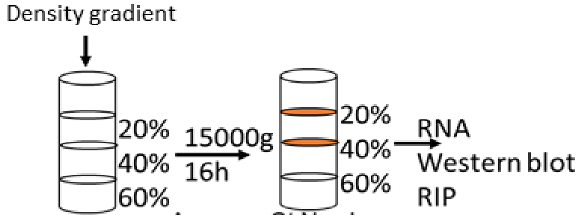

Average Ct Number

EVs Fractions	miR-320a	SD	miR-125b	miR-34a	SD±
WT-20/40	38.2	0.17	39.8	34.74	0.94
WT/40/60	44.8	0.28	43.6	44.93	N/A
CD47-20/40	36.7	0.03	40.2	35.27	0.64
CD47-/40/60	N/A	N/A	N/A	N/A	N/A

C

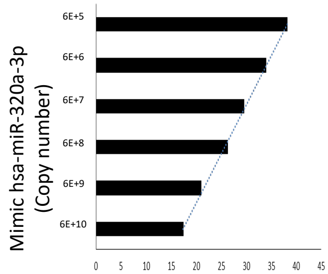

D

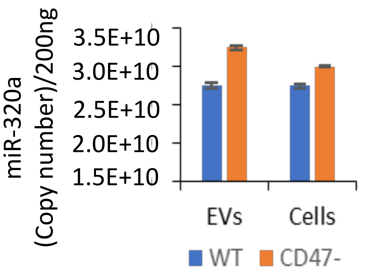

E

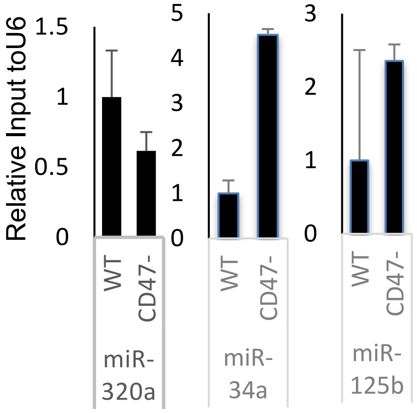

F

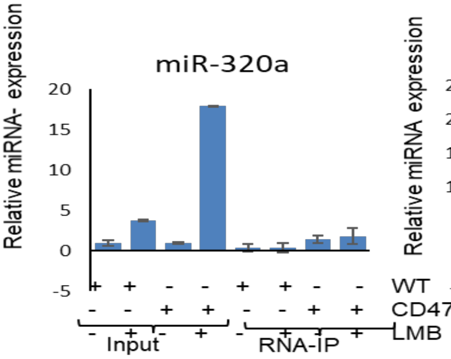

G

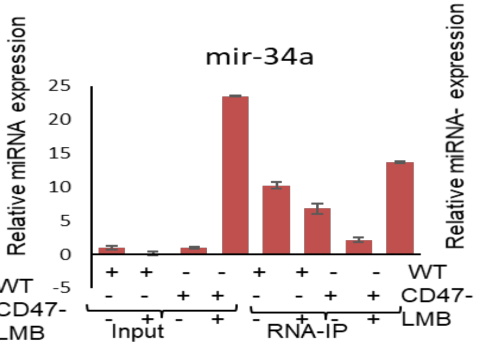

H

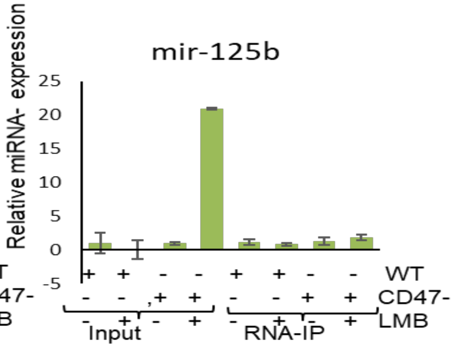

Supplemental Figure S6

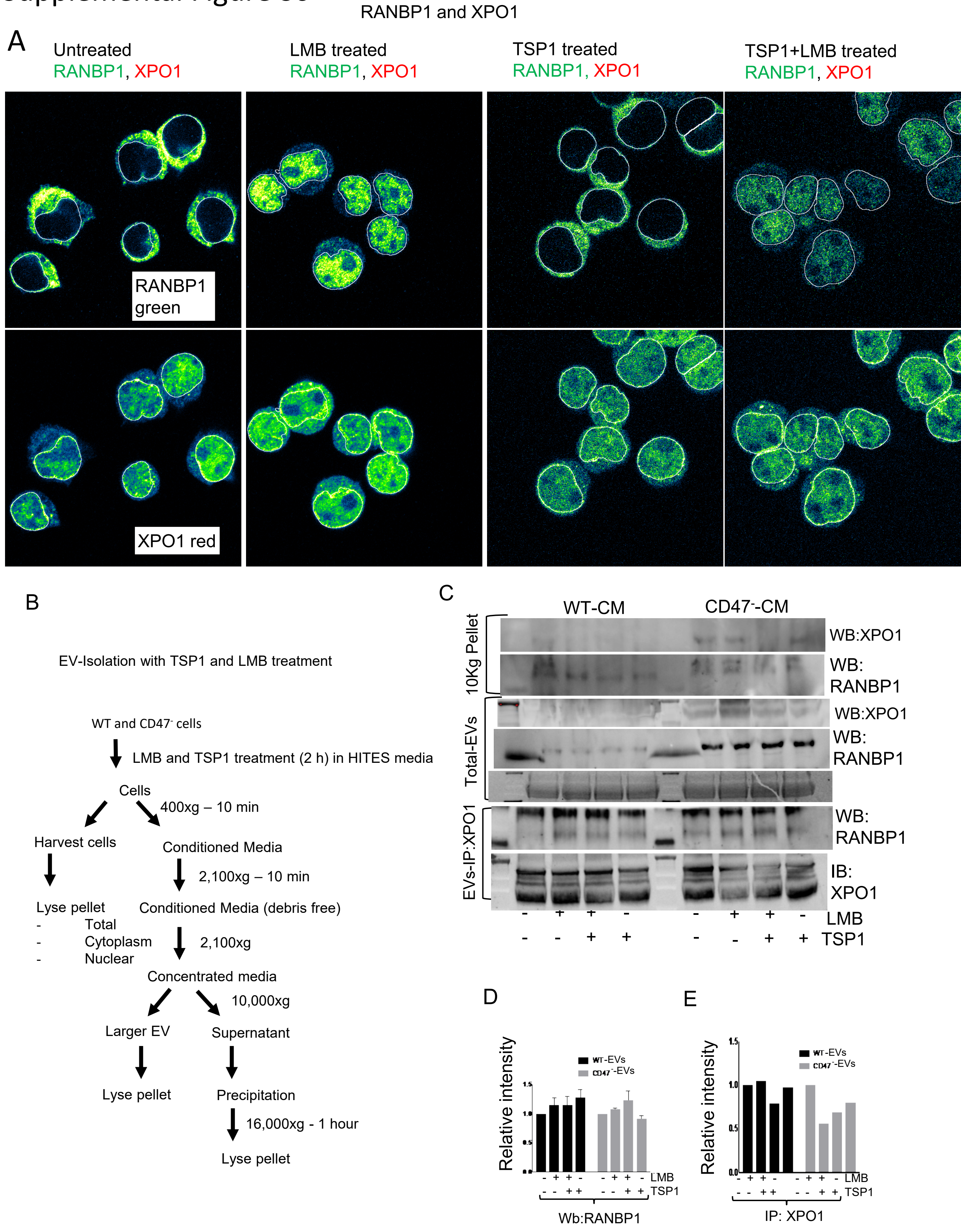

Supplemental Figure S7 (Table S1)

Name	Sequence
U 6-Forward	CTCGCTTCGGCAGCACA
U 6-Reverse	AACGCTTCACGAATTTGCGT
Pre-miR103-1,2-Forward	GCTTCTTTACAGTGCTGCCT
Pre-miR103-1,2-Reverse	TTCATAGCCCTGTACAATGCT
Pre-miR107-Forward	CAGCTTCTTTACAGTGTTGCCT
Pre-miR107-Reverse	GATAGCCCTGTACAATGCTGC
Pre-miR34a-Forward	TGGCAGTGTCTTAGCTGGTTG
Pre-miR34a-Reverse	GGCAGTATACTTGCTGATTGCTT
Pre-miR125b-1-Forward	GTCCCTGAGACCCTAACTTG
Pre-miR125b-1-Reverse	AGCCTAACCCGTGGATTT
let 7a-3-Forward	AGA ATC CCT GTG CCC TTG G
let 7a-3-Reverse	GGC ACC TAG GCC TGT CAG ACT
Pre-miR15b-Forward	AGCACATCATGGTTTACATGC
Pre-miR15b-Reverse	CTAGAGCAGCAAATAATGATTCG
Pre-miR138-1,2-Forward	CAGCTGGTGTTGTGAATCAG
Pre-miR138-1,2-Reverse	ACCCTGGTGTCGTGAAATAG
Pre-miR191-Forward	GCAACGGAATCCCAAAAG
Pri-miR31-Forward	TGAGTGTGTTTTCCCTCCCT
Pri-miR31-Reverse	GCCATGGCTGCTGTCAG
Pre-miR191-Reverse	GACGAAATCCAAGCGCA
18S r RNA-F	AGG ACC GCG GTT CTA TTT TGT TGG
18S rRNA-R	CCC CCG GCC GTC CCT CTT A
B2M-F	TCC TGA ATT GCT ATG TGT CTG GGT
B2M-R	GAT AGA AAG ACC AGT CCT TGC T
ETS1-F	TCATTTCTTTGCTGCTTGGA
ETS1-R	CTCACCATCATCAAGACGGA
EEF1A1-F	ACTTGCCCGAATCTACGTGT
EEF1A1-R	TTGCCGCCAGAACACAG
YOD1-F	CAATGGGGATACCATTCTGG
YOD1-R	ACCACGGTTCTGGTAAGCAC
Eef1a1 Mouse-F	ACGAGGCAATGTTGCTGGTGAC
Eef1a1 Mouse-R	GTGTGACAATCCAGAACAGGAGC
Ets1-mouse-F	CCAGAATCCTGTTACACCTCGG
Ets1-mouse-R	CAGCGTCTGATAGGACTCTGTG
Yod1-mouse-F	GTCAGCGAATCCTCGTTGGCTA
Yod1-mouse-R	CGCAGGTGAAGCTTTTGGTCTG
XPO1-F	GACGTCTTGATAATGAGGCCA
XPO1-R	TTGGAAAATGTGATAAAAACAAGG
GAPDH-F	AGTATGACAACAGCCTCAAGA
GAPDH-R	GTCCTTCCACGATACCAAA
